# Pyrene‐linked heterocyclic pyrazoles: Synthesis, antimicrobial screening, and in silico molecular insights

**DOI:** 10.1002/smo2.70074

**Published:** 2026-07-28

**Authors:** Dinkal V. Kasundra, Paresh N. Patel

**Affiliations:** ^1^ Laboratory of Bio‐Organic Chemistry Tarsadia Institute of Chemical Science (TICS) Uka Tarsadia University Bardoli Gujarat India

**Keywords:** antimicrobial screening, heterocyclic, molecular docking, pyrazoles, pyrene

## Abstract

A library of structurally diverse pyrene‐linked pyrazole derivatives was designed and synthesized, and their antimicrobial properties along with molecular interaction behavior were systematically investigated. The target compounds were efficiently prepared through a straightforward synthetic approach and fully characterized using standard spectroscopic techniques, confirming the formation of the desired heterocyclic framework. Biological evaluation against selected Gram‐positive, Gram‐negative, and fungal strains showed noticeable differences in activity across the series, indicating a strong dependence on the nature and position of substituents as well as their electronic characteristics. Notably, compound 4h emerged as the most active derivative, exhibiting significant antibacterial effects with inhibition zones of approximately 38 mm against *Staphylococcus aureus* and 29 mm against *Proteus vulgaris*, demonstrating broad‐spectrum potential. To better understand these results, molecular docking studies were carried out against the *Escherichia coli* FabB enzyme. Compound 4 h showed the most favorable binding energy (Δ*G* = −8.9 kcal/mol), supported by stable hydrogen bonding and hydrophobic interactions within the enzyme's active site. In silico analysis also revealed a cLogP value of 7.3390, indicating suitable lipophilicity consistent with drug‐like behavior. In summary, the integration of experimental antimicrobial assessment with computational studies supports the pyrene–pyrazole framework as a promising scaffold for the further development of effective antimicrobial agents.

## Graphical abstract

A series of novel pyrene‐linked heterocyclic pyrazoles were synthesized and structurally characterized. The compounds were evaluated for antimicrobial activity against selected bacterial and fungal strains, showing promising inhibitory effects. Molecular docking studies provided insights into binding interactions and structure–activity relationships, supporting their potential as antimicrobial lead candidates.

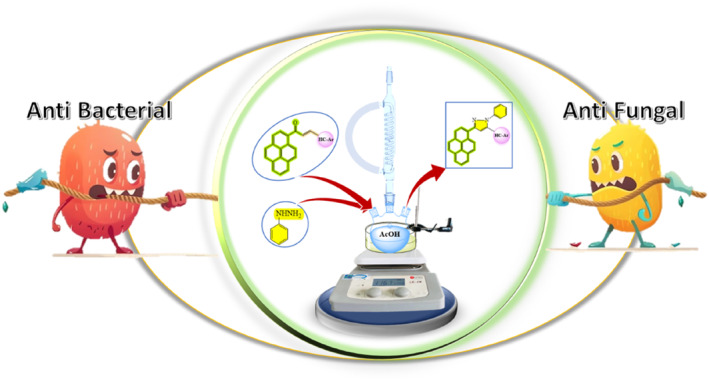

## 1 INTRODUCTION

Heterocyclic compounds have consistently occupied a prominent position in medicinal chemistry due to their structural versatility and broad biological relevance.^[1]^ Among these, pyrazole derivatives have received significant attention because of their diverse pharmacological properties.^[2]^ Numerous studies have reported their antimicrobial, anti‐inflammatory, and anticancer activities.[^3^, ^4^, ^5^] The presence of two adjacent nitrogen atoms in the pyrazole ring enables effective hydrogen bonding and coordination with biological targets, which enhances their therapeutic potential.[^6^, ^7^, ^8^]

Pyrene is a polycyclic aromatic hydrocarbon characterized by a rigid planar structure and extended π‐conjugation.^[9]^ It has been extensively utilized in materials science and bioactive molecular design.^[10]^ Previous investigations have shown that incorporation of a pyrene unit can improve lipophilicity, membrane permeability, and π–π stacking interactions.^[11]^ These properties facilitate stronger binding with hydrophobic regions of proteins and nucleic acids.^[12]^ Consequently, pyrene‐based conjugates have demonstrated promising biological activities in earlier reports.^[13]^

The increasing prevalence of antimicrobial resistance has become a major global health concern.^[14]^ Many conventional antibiotics are losing effectiveness due to resistance mechanisms such as target modification and enzymatic degradation.^[15]^ This situation demands the development of structurally novel and mechanistically distinct antimicrobial agents. In recent years, hybrid molecular design has emerged as a rational strategy to enhance biological efficacy.^[16]^ Combining two pharmacologically active scaffolds within a single framework often leads to improved potency and selectivity.^[17]^

**FIGURE 1:**
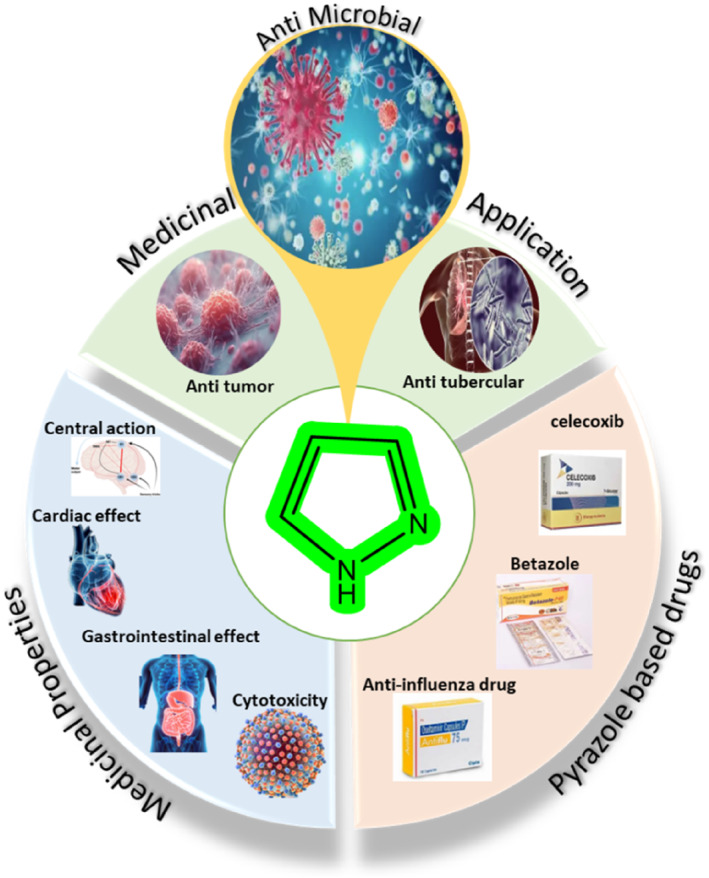
Graphical depiction of the pyrazole core and its role in pharmaceutical and biomedical research.

Pyrene‐appended compounds have shown promising antimicrobial potential, where structural modification enhances interaction with microbial targets.^[18]^ Pyrazole‐linked aromatic systems are also well known for broad‐spectrum antimicrobial properties.^[19]^ In parallel, molecular docking studies suggest strong binding of such scaffolds to key bacterial enzymes such as DNA gyrase through π–π stacking and hydrophobic interactions.^[20]^ However, systematic studies correlating the structure and antimicrobial activity of pyrene‐appended pyrazoles remain limited (Figure 1).

The integration of pyrene and pyrazole motifs represents a logical and innovative approach in this direction.^[21]^ The pyrene fragment provides hydrophobic and aromatic interactions, while the pyrazole ring contributes hydrogen‐bonding capability and electronic adaptability.^[22]^ Earlier studies have suggested that such structural combinations can enhance binding affinity toward microbial targets and improve biological performance.^[23]^

In addition to experimental evaluation, computational approaches have become integral to modern drug discovery.^[24]^ Molecular docking helps predict ligand–protein interactions and provides insight into possible mechanisms of action.^[25]^ These methods support structure–activity relationship (SAR) analysis and rational molecular optimization.^[26]^

In the present study, novel pyrene‐based heterocyclic pyrazole derivatives were designed and synthesized using an efficient synthetic route. The synthesized compounds were characterized using standard spectroscopic techniques and evaluated for antimicrobial activity against selected bacterial strains. Furthermore, in silico investigations were conducted to explore their binding interactions and electronic properties. This integrated experimental and computational study aims to identify promising candidates for future antimicrobial development.

## 2 EXPERIMENTAL DETAILS

### 2.1 Synthesis of pyrene‐heterocyclic scaffolds from 1 acetyl pyrene (3a–3h)

All the pyrene‐heterocyclic DAES were synthesized following our previously reported procedure.^[27]^ In a typical reaction, 1‐ Acetyl pyrene (**1**; 0.122 g, 0.5 mmol) was dissolved in a minimal amount of ethanol (3 mL) in a 25 mL round‐bottom flask and stirred to obtain a clear solution. To this, 2‐Pyridinecarbaldehyde (**2a**; 47.55 μL, 0.5 mmol) was added, and the mixture was stirred for 5 min at room temperature. A catalytic amount of KOH or pyrrolidine solution in ethanol (4%, 2 mL) was then introduced dropwise under continuous stirring. The reaction mixture was allowed to stir further at room temperature for 2–3 h in the presence of KOH or 5–6 h when pyrrolidine was used. Progress of the reaction was checked by thin‐layer chromatography (TLC). As the reaction proceeded, the product gradually precipitated from the mixture. After completion, the solid product was cooled, filtered, washed with cold ethanol, and purified by recrystallization using a chloroform–methanol mixture (1:1 v/v, 8 mL). **3a** was obtained as a yellow solid with yields of 74% (2.45 g, 2 h) using KOH and 87% (2.79 g, 6 h) using pyrrolidine. The same protocol was applied for the preparation of other derivatives (**3b–3 h**) by substituting appropriate aldehydes (**2b–2 h**).

### 2.2 General method for the synthesis of pyrazoles (4a–4h) from pyrene‐heterocyclic DAES

**SCHEME 1:**
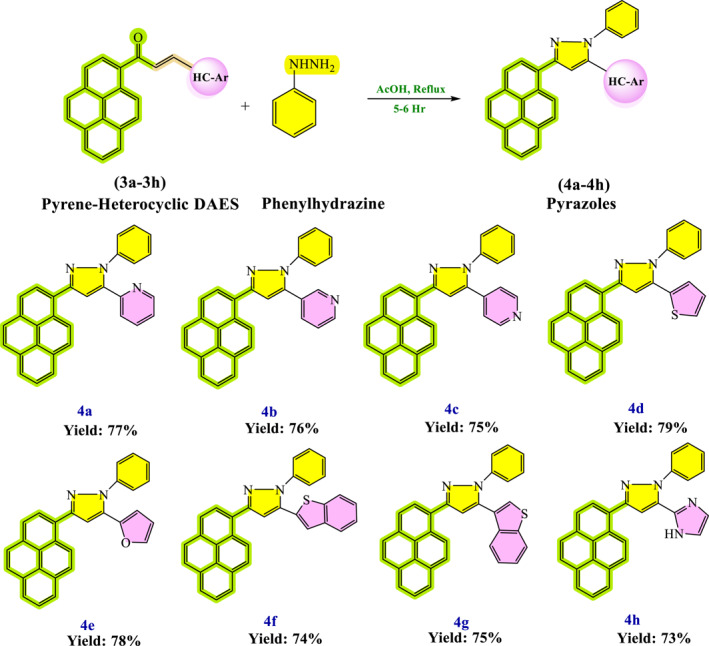
Synthetic strategy of pyrazole derivatives from 1‐acetyl pyrene‐based DAES.

A solution of the corresponding DAES (3a–3h, 1 mmol) was prepared in a small quantity of glacial acetic acid until complete dissolution was achieved. Phenyl hydrazine (2.3 g, 3 mmol) was then introduced, and the reaction mixture was heated under reflux for 5–6 h. Reaction progress was periodically checked by TLC using an ethyl acetate/n‐hexane (1:3) system as the eluent. After completion, the mixture was allowed to cool and poured into crushed ice, resulting in the formation of a solid precipitate. The obtained solid was collected by filtration, washed thoroughly with cold water, and dried under reduced pressure (Scheme 1).

The crude material was purified by column chromatography over silica gel (70–230 mesh). A mixture of ethyl acetate and dichloromethane (1:4, v/v) was employed as the mobile phase. The purified product was obtained after column chromatography to afford the desired pyrazole derivatives (4a–4h) in good yields.

### 2.3 Detail mechanism for the synthesis of pyrazoles (4a–4h)

The formation of pyrene‐linked pyrazole derivatives proceeds via initial condensation between the corresponding pyrene‐functionalized chalcone (α, *β*‐unsaturated carbonyl system) and phenyl hydrazine. The nucleophilic attack of hydrazine on the carbonyl carbon leads to the formation of a phenyl hydrazone intermediate, followed by Michael‐type addition across the activated double bond. Subsequent intramolecular cyclization and proton transfer steps result in the formation of the pyrazoline intermediate, which undergoes oxidative aromatization under reaction conditions to furnish the final pyrazole ring system. The presence of the electron‐rich hydrazine facilitates cyclization, while conjugation with the pyrene moiety stabilizes intermediates through π‐electron delocalization.

### 2.4 Biological assessment

**Antibacterial susceptibility testing of the synthesized pyrazoles (4a–4h)**: The antimicrobial efficacy of the synthesized pyrazoles was measured against two Gram‐positive (*Staphylococcus aureus*, *Bacillus subtilis*) and two Gram‐negative (*Escherichia coli*, *Pseudomonas vulgaris*) bacterial and two fungal strains *Aspergillus flavus* and *Aspergillus Niger*. Bacterial cultures were prepared in Brain Heart Infusion broth and incubated at 37°C for 6 h to achieve logarithmic growth. Each microorganism was then adjusted to a concentration of 10^6^ colony‐forming units/mL. Aliquots (100 μL) of the inoculum were uniformly spread onto the surface of Mueller‐Hinton agar plates using a sterile spreader.

Sterile filter paper discs (6 mm diameter) were saturated with 25 and 50 μg of each test compound dissolved in dimethyl sulfoxide (DMSO) and placed onto the protected agar surfaces. For comparative analysis, wells (6 mm diameter) were aseptically created in fresh agar plates, into which 25 and 50 μg of the compounds were directly introduced. Dimethyl sulfoxide alone served as a negative control, and ciprofloxacin was used as a positive control due to its broad‐spectrum antibacterial activity at concentrations of 25 and 50 μg per well. Its well‐established clinical relevance, allowing reliable comparison of the antibacterial efficacy of the synthesized pyrazoles.

All plates were incubated at 37°C for 24 h. Post incubation, the diameters of the inhibition zones were measured in millimeters using a digital caliper. A zone diameter of 7 mm or greater was considered indicative of susceptibility. Each experiment was conducted in triplicate to ensure reproducibility.

**Antifungal susceptibility testing of the synthesized pyrazoles (4a‐4h)**: The fungal strain was cultured on Sabouraud Dextrose Agar (SDA) and incubated at 37°C for 24 h. A loopful of the resulting culture was suspended in sterile 0.85% saline solution and vortexed to achieve a uniform suspension. The turbidity of the suspension was adjusted to match the 0.5 McFarland standard, corresponding to approximately 1–5 × 10^6^ cells/mL, by visually comparing the suspension against a white background with contrasting black lines. A sterile cotton swab was dipped into the adjusted fungal suspension, excess liquid was removed by pressing the swab against the tube wall, and the swab was then used to evenly inoculate the surface of SDA plates by streaking in three perpendicular directions to ensure uniform distribution. The inoculated plates were allowed to stand for 15 min to facilitate initial attachment of the fungal cells to the agar surface.

Using a sterile 6 mm cork borer, wells were aseptically created in the inoculated agar plates. Each well was filled with 25 and 50 μg of the test compound dissolved in DMSO, with DMSO alone serving as a negative control. The plates were then refrigerated at 4°C for 2 h to allow for diffusion of the compounds into the agar medium. Subsequently, the plates were incubated at 37°C for 24 h.

After incubation, the diameters of the zones of inhibition were measured in millimeters using a ruler. Ketoconazole was used as a positive control at concentrations of 25 and 50 μg per well. All experiments were performed in triplicate to ensure reproducibility and reliability of the results.

Antibacterial and antifungal studies were conducted at Shristi Laboratory. The microbial strains used in this study were maintained and provided by the same laboratory under standard microbiological conditions. All antibacterial and antifungal assays were conducted in this laboratory following established protocols to ensure accuracy and reproducibility of the results.

**Determination of minimum inhibitory concentration of the synthesized pyrazoles (4a–4h)**: Minimum inhibitory concentration (MIC) values of the synthesized compounds were determined in biological triplicates using the agar dilution method against selected bacterial and fungal strains. Antibacterial activity was evaluated against Gram‐positive (*S*. *aureus*, *B*. *subtilis*) and Gram‐negative (*E*. *coli*, *P*. *vulgaris*) bacteria, while antifungal activity was assessed against *A*. *flavus* and *A*. *Niger*. Stock solutions were prepared in DMSO and serially diluted to obtain the required concentrations.

Mueller–Hinton agar (for bacteria) and SDA (for fungi) were incorporated with different concentrations of the test compounds and poured into sterile Petri plates. Standardized microbial inoculates were spot‐inoculated onto the agar surface. Ciprofloxacin and ketoconazole were used as reference antibacterial and antifungal drugs, respectively. Plates were incubated at 37°C for 18–24 h (bacteria) and 28–30°C for 48–72 h (fungi). The MIC was defined as the lowest concentration showing no visible microbial growth.

**Statistical analysis**: All experimental data were expressed as mean ± standard deviation (SD) from at least three independent measurements (*n* ≥ 3). Statistical analysis was performed using software Origin. Significant differences between groups were evaluated using one‐way analysis of variance (ANOVA), followed by an appropriate post hoc test (Tukey's multiple comparison test). A *p*‐value of less than 0.05 was considered statistically significant.

### 2.5 Computational study

**Molecular docking studies**: The antibacterial activity of the pyrazole derivatives was investigated through molecular docking studies using AutoDock Vina and AutoDock Tools. The docking workflow involved three main steps: protein preparation, ligand preparation, and docking simulation.^[28]^ The ATPase domain of *Escherichia coli* DNA gyrase B **(PDB ID: 4DUH)** was selected as the target protein due to its essential role in bacterial DNA replication.^[29]^ The crystal structure was obtained from the RCSB Protein Data Bank (https://www.rcsb.org/structure/4DUH) and prepared by removing water molecules and co‐crystallized ligands, followed by the addition of polar hydrogens and assignment of Gasteiger charges. A cubic grid box of **20 × 20 × 20 Å** was defined around the active site to confine the docking region.

Ligand structures were generated in 3D format using AutoDock Tools, followed by energy minimization, application of Gasteiger charges, merging of polar hydrogens, and definition of rotatable bonds to obtain PDBQT files.[^29^, ^30^] Docking simulations were performed using AutoDock Vina with predefined grid coordinates and exhaustiveness parameters.^[31]^ The resulting binding poses were ranked based on predicted binding affinities (kcal/mol), and the most favorable conformations were analyzed using Discovery Studio Visualizer to identify key hydrogen bonding and hydrophobic interactions with active‐site residues.^[32]^

The docking protocol was validated by redocking the native inhibitor, which reproduced its binding orientation within the active site with an RMSD <3.0 Å, confirming the reliability of the docking approach. These results provide valuable insight into the binding behavior of the synthesized pyrazole derivatives against DNA gyrase B and support their potential as antibacterial agents.

## 3 RESULTS AND DISCUSSION

### 3.1 Reaction condition optimization for pyrazoles synthesis

The pyrazole derivatives were synthesized by reacting phenyl hydrazine with our previously reported fluorochromes[^33^, ^34^, ^35^, ^36^] through a cyclization reaction facilitated by acetic acid. The influence of various solvents, temperatures and reaction durations on the formation of pyrazole derivatives was systematically examined, with the findings summarized in Table 1.

**TABLE 1:** Reaction condition optimization for pyrazole production.

Sr. No.	Solvent	Phenyl hydrazine (mole equivalent)	Temperature (°C)	Time (hr)	Yield (% conversion)
1	Benzene	1	RT (∼30)	5	No reaction
2	THF	1	RT (∼30)	5	No reaction
3	Ethanol	1	RT (∼30)	5	No reaction
4	Acetic acid	1	RT (∼30)	5	7
5	Acetic acid	1	Reflux	3	18
6	Acetic acid	1	Reflux	5	59
7	Acetic acid	2	RT (∼30)	5	32
8	Acetic acid	2	Reflux	5	66
9	Acetic acid	3	RT (∼30)	5	46
10	Acetic acid	3	Reflux	5	79

The synthesis of pyrazole derivatives was systematically optimized by evaluating the influence of various solvents, temperatures, and reactant ratios. Initial attempts using non‐polar solvents like benzene and aprotic solvents such as tetrahydrofuran at room temperature yielded no product even after 5 h, indicating their ineffectiveness for this reaction. Subsequent trials with ethanol resulted in product formation but with low conversion rates. In contrast, conducting the reaction in acetic acid under reflux conditions significantly enhanced the yield, achieving substantial conversion in 5 h.

Further optimization revealed that employing a 3:1 M ratio of phenyl hydrazine to the derivative **(3a–3h)** under reflux conditions provided the highest yield, with a 79% conversion rate. This optimized method was successfully applied to synthesize a series of pyrazole derivatives **(4a–4h)**, all exhibiting yields between 70% and 77%. These results underscore the efficacy of acetic acid as a solvent in facilitating the cyclization of derivatives to pyrazoles. The established reaction conditions offer a reliable and efficient approach for synthesizing pyrazole derivatives with consistent yields.

### 3.2 Spectroscopic characterization of synthesized pyrazoles (4a–4h)

The novel synthesized pyrazole derivatives **(4a–4h)**, were thoroughly characterized using various analytical techniques to confirm their structures **(**Supporting Information S1; Figures S1–S24). The FT‐IR spectra displayed a significant absorption band around 1600–1630 cm^−1^, corresponding to the C=N stretching vibration, which is characteristic of the pyrazole ring. Additional medium‐intensity bands observed in the region of 3012–2876 cm^−1^ were attributed to the C–H stretching vibrations of the aromatic and aliphatic groups, further supporting the formation of the pyrazole structure. The disappearance of the α, *β*‐unsaturated carbonyl group absorption (1600–1700 cm^−1^) and the appearance of a band indicative of –CH_2_– groups also corroborated the successful cyclization to pyrazole derivatives. The ^1^H NMR spectra revealed characteristic signals, including a doublet of doublets around 8.425 ppm (*J* = 15.3 and 7.9 Hz), corresponding to the –CH_2_– group adjacent to the pyrazole ring. A doublet at 8.653 ppm (*J* = 9.3 Hz) was observed for the –CH– proton attached to the pyrazole ring. The aromatic region (6–8 ppm) displayed multiplets corresponding to the aromatic protons, consistent with the expected substitution pattern. The ^13^C NMR spectra exhibited signals in the range of 110–140 ppm, attributed to the aromatic carbons. Downfield shifts around 145–146 ppm were assigned to the C=N and C–N carbons within the pyrazole ring. High‐resolution mass spectrometry analysis provided molecular ion peaks consistent with the expected molecular masses of the compounds, supporting their molecular formulas as hydrogen adduct (M + H).

Collectively, these spectroscopic data confirm the successful synthesis and structural integrity of the pyrazole derivatives.

### 3.3 Comparative antimicrobial activity of synthesized pyrazoles

The antibacterial potential of pyrazole derivatives (**4a–4h**) was assessed against four strains—*S*. *aureus*, *B*. *subtilis*, *P*. *vulgaris*, and *E*. *coli*—with inhibition zone values summarized in Table 2.

**TABLE 2:** Antibacterial efficacy of synthesised pyrazole.

Sample	Zone of inhibition in millimeter (mm)							
	Gram‐positive				Gram‐negative			
	*S*. *Aureus* (ATCC‐19433)		*B*. *Subtilis* (ATCC‐6633)		*E*. *Coli* (ATCC‐ 8739)		*P*. *Vulgaris* (ATCC‐29213)	
	25 μg/well	50 μg/well	25 μg/well	50 μg/well	25 μg/well	50 μg/well	25 μg/well	50 μg/well
4a	32 ± 2	35 ± 2	31 ± 2	32 ± 2	19 ± 2	22 ± 2	24 ± 2	33 ± 2
4b	30 ± 2	32 ± 1	27 ± 1	31 ± 1	17 ± 1	20 ± 1	21 ± 2	23 ± 1
4c	33 ± 2	36 ± 2	30 ± 2	34 ± 2	20 ± 2	24 ± 2	27 ± 2	36 ± 2
4d	31 ± 2	34 ± 1	30 ± 1	33 ± 1	18 ± 2	22 ± 2	24 ± 1	25 ± 2
4e	26 ± 2	29 ± 2	26 ± 2	29 ± 2	17 ± 1	21 ± 1	21 ± 2	24 ± 2
4f	22 ± 2	26 ± 1	24 ± 1	27 ± 1	16 ± 2	20 ± 1	18 ± 1	21 ± 1
4g	29 ± 1	31 ± 2	27 ± 1	31 ± 2	17 ± 1	20 ± 1	21 ± 2	23 ± 1
4h	**38 ± 1**	**41 ± 1**	**34 ± 1**	**37 ± 1**	**24 ± 2**	**28 ± 2**	**31 ± 1**	**40 ± 1**
Ciprofloxacin	35 ± 2	37 ± 2	32 ± 2	36 ± 2	22 ± 2	26 ± 2	29 ± 2	38 ± 2
DMSO	‐	‐	‐	‐	‐	‐	‐	‐

*Note*: Bold values indicate the highest activity among all tested compounds.

The zones of inhibition observed for the synthesized compounds were measured and directly compared with those produced by the respective standard drugs, ciprofloxacin for antibacterial activity and ketoconazole for antifungal activity, tested under identical experimental conditions.

Overall, the compounds showed moderate efficacy, yet fell short compared to standard antibiotics.^[37]^ Among the Gram‐positive bacteria, **4h** stood out for its strong activity against *S*. *aureus*, with inhibition zones of approximately 38 mm, while the rest (**4a–4f**) produced zones ranging from 22 to 33 mm. Notably, pyrazole **4h** achieved the largest zone, likely due to its multi‐heteroatom framework and lower π‐conjugation, which may enhance dual binding interactions with bacterial targets.

Against Gram‐negative bacteria, compound **4h** demonstrated especially strong activity, producing an inhibition zone of approximately 31 mm against *P*. *vulgaris*. Additionally, both **4h** showed noteworthy effects on *E*. *coli*, with zones measuring about 24 mm. These outcomes underscore the capability of this pyrazole‐based compound to act effectively against both Gram‐positive and Gram‐negative pathogens, hinting at their potential as versatile antimicrobial agents.

Although the pyrazole derivatives showed moderate antibacterial activity, they didn't match the potency of standard antibiotics, highlighting the need for further optimization to improve their effectiveness. Using DMSO as the negative control confirmed that the antibacterial effects observed restricted only from the compounds rather than the solvent.

We evaluated the antifungal activity of all synthesized compounds against two fungal strains. The results demonstrated strong to excellent antifungal effects across the board. Notably, pyrazole **4h** showed the strongest activity against *A*. *flavus*, producing a 40 mm inhibition zone—comparable to standard antifungal agents (Table 3). The other compounds (**4a–4f**) also showed considerable activity, with inhibition zones ranging from 26 to 35 mm. The pronounced effectiveness of pyrazole **4h** is likely linked to its increased lipophilicity, which may enhance its penetration and interaction with fungal cell targets.

**TABLE 3:** Antifungal efficacy of synthesised pyrazole.

Sample	Zone of inhibition (mm)			
	*Aspergillus flavus* (MTCC‐1884)		*Aspergillus niger* (MTCC‐1881)	
	25 μg/well	50 μg/well	25 μg/well	50 μg/well
4a	33 ± 2	37 ± 2	30 ± 3	34 ± 2
4b	32 ± 2	36 ± 2	29 ± 2	32 ± 1
4c	35 ± 2	38 ± 3	31 ± 1	34 ± 2
4d	34 ± 1	38 ± 1	28 ± 3	34 ± 1
4e	28 ± 1	32 ± 1	23 ± 1	26 ± 1
4f	26 ± 1	30 ± 1	21 ± 1	25 ± 1
4g	30 ± 1	33 ± 1	25 ± 1	27 ± 1
4h	**40 ± 1**	**45 ± 1**	**35 ± 1**	**40 ± 1**
Ketoconazole	43 ± 1	46 ± 1	37 ± 2	42 ± 1
DMSO	‐	‐	‐	‐

*Note*: Bold values indicate the highest activity among all tested compounds.

The findings from this study validate the antibacterial and antifungal efficacy of the synthesized pyrazole derivatives and lay a strong groundwork for future investigations. Enhancing their potency through structural optimization and broadening the antimicrobial scope especially paired with in vivo studies could cover the way for new treatments against bacterial and fungal infections. The promising activity against diverse pathogens also highlights the potential of these compounds to fight the growing threat of antimicrobial resistance.^[38]^

The synthesized compounds exhibited notable antimicrobial activity with strain‐dependent MIC values (Table 4). Against Gram‐positive bacteria, *S*. *aureus*, good antibacterial activity was observed with MIC values of **7.02 μg/mL**, comparable to ciprofloxacin in some cases. For Gram‐negative strains, *P*. *vulgaris*, moderate activity was recorded, with MIC values **12.54 μg/mL**, likely due to reduced membrane permeability. The pyrazole derivatives exhibited varying degrees of antimicrobial activity against both Gram‐positive and Gram‐negative bacterial strains as well as fungal species. While the reference drug ciprofloxacin showed superior activity with MIC values in the sub‐micromolar range, several synthesized compounds demonstrated MIC values within the low‐to‐moderate micromolar range, which is considered acceptable for early‐stage antimicrobial lead compounds. Similarly, antifungal evaluation against *A*. *flavus* and *Aspergillus niger* revealed moderate activity compared to ketoconazole, indicating that the biphenyl‐pyrazole scaffold provides a promising structural framework for further optimization.

**TABLE 4:** Strain‐dependent MIC values of synthesised pyrazole.

Compound	Gram‐positive bacteria (μg/mL)		Gram‐negative bacteria (μg/mL)		Fungi (μg/mL)	
	*Staphylococcus aureus*	*Bacillus subtilis*	*Escherichia coli*	*Proteus vulgaris*	*Aspergillus flavus*	*Aspergillus niger*
4a	8.71	8.83	13.47	13.56	14.25	26.24
4b	8.90	8.99	13.63	13.74	14.36	26.39
4c	7.87	7.98	12.59	12.67	13.83	25.80
4d	8.21	8.34	12.91	13.04	13.97	25.92
4e	9.12	9.22	14.16	14.27	15.09	27.04
4f	10.25	10.68	15.75	15.54	16.51	16.00
4g	10.87	10.98	16.59	16.67	17.83	17.80
4h	**7.02**	**7.68**	**12.75**	**12.54**	**13.31**	**24.40**
Ciprofloxacin	0.25	0.25	0.5	0.5	—	—
Ketoconazole	—	—	—	—	0.5	0.5

*Note*: Bold values indicate the highest activity among all tested compounds.

Abbreviation: MIC, minimum inhibitory concentration.

Antifungal screening showed promising activity against *A*. *flavus* (**13.31 μg/mL**), while moderate inhibition was observed against *Aspergillus niger* with MIC values of **24.40 μg/mL**, with pyrazole **4h** approaching the activity of ketoconazole. The reported MIC values represent reproducible end‐point results obtained across independent experiments.

Due to the discrete end‐point nature of MIC determination, formal statistical tests (ANOVA or *t*‐test) were not applied; instead, biological triplicates were performed to ensure reproducibility, and results are expressed as mean ± SD.

### 3.4 Molecular study assessment

**FIGURE 2:**
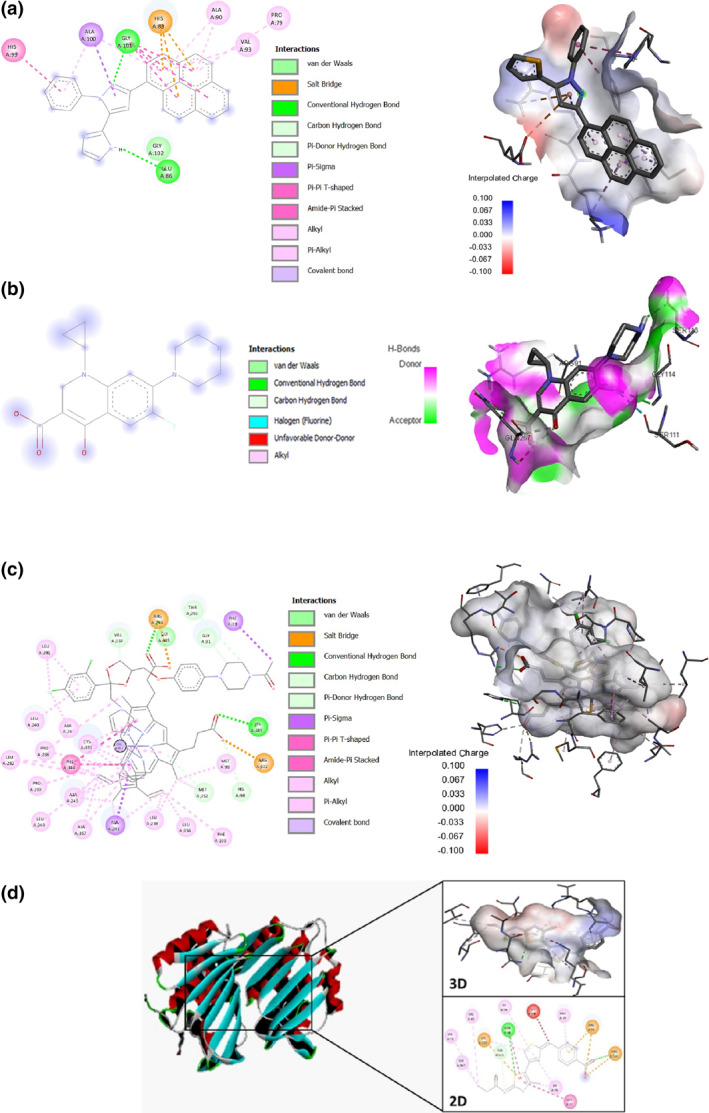
(a) Two‐ and three‐dimensional binding orientations of docked pyrazole 4h. (b) Molecular docking analysis of ciprofloxacin (CIP) with the active site of DNA gyrase (2D and 3D views). (c) Binding interaction of ketoconazole with the active site of pyrazole 4h. (d) Protein–ligand interaction diagrams (2D and 3D) showing the binding site binding within the ATPase domain of DNA gyrase B (PDB ID: 4DUH).

The molecular docking results of the synthesized pyrazoles against the ATPase domain of *Escherichia coli* DNA gyrase B (PDB ID: 4DUH) are tabulated in Table 5 and illustrated in Figure 2. All pyrazoles (Supporting Information S1; Figures S25–S31) exhibited effective binding within the ATP‐binding pocket, with several derivatives showing favorable binding energy values comparable to the reference drugs ciprofloxacin and ketoconazole. The 3D docking poses demonstrate stable accommodation of the ligands inside the active site, whereas the corresponding 2D interaction diagrams clearly depict the key ligand–protein interactions.

**TABLE 5:** Binding affinities and interaction profiles of 4a–4h, ciprofloxacin, and ketoconazole within the ATPase domain of DNA gyrase B (PDB ID: 4DUH).

Sr. No.	Binding energy (kcal/mol)	Interacting residues
4a	−9.4	GLU(A:86), GLU(A:85), GLY(A:102), GLY(A:101), ALA(A:100), ALA(A:90), HIS(A:99), HIS (A: 83) VAL (A:93), PRO(A;79)
4b	−9.5	GLU(A:86), GLU(A:85), GLY(A:102), GLY(A:101), ALA(A:100), ALA(A:90), HIS(A:99), HIS (A: 83) VAL (A:93), PRO(A;79)
4c	−9.6	VAL(A:118), PHE(A:41), HIS(A:38), ARG(A:190), LYS(A:189), ILE(A:186)
4d	−10.0	PHE(A:41), HIS(A:38), ARG(A:190), LYS(A:189), ILE(A:186)
4e	−9.4	VAL(A:118), GLU(A:42), HIS(A:38), ARG(A:190), LYS(A:189), ILE(A:186)
4f	−11.1	GLU(A:85), GLY(A:102), GLY(A:101), ALA(A:100), ALA(A:90), HIS(A:99), HIS (A: 83) VAL (A:93), VAL (A:97), PRO(A;79)
4g	−11.0	GLU(A:85), GLY(A:102), GLY(A:101), ALA(A:100), ALA(A:90), HIS(A:99), HIS (A: 83) VAL (A:93), VAL (A:97), PRO(A;79)
4h	**−8.9**	**GLU(A:85), GLY(A:102), GLY(A:101), ALA(A:100), ALA(A:90), HIS(A:99), HIS (A: 83) VAL (A:93), VAL (A:97), PRO(A;79)**
Ciprofloxacin	−13.2	VAL(A:1045), ARG(B:1033), HIS(B:1081), LYS(B:460), ASP(B:908), ASP(B:510), TYR(B:1150), PRO(B:1080)
Ketoconazole	−13.2	VAL(A:1045), ARG(B:1033), HIS(B:1081), ASP(B:908), TYR(B:1150), PRO(B:1080)

Interaction analysis revealed hydrogen bonding with crucial residues such as **ARG(A:190), HIS(A:38), and LYS(A:189),** along with hydrophobic interactions involving **ILE(A:186)** which is known to play an essential role in ATP binding and enzyme inhibition. The most active compound displayed a binding mode closely resembling that of ciprofloxacin, indicating a similar interaction pattern within the ATPase domain. In contrast, ketoconazole exhibited additional hydrophobic stabilization within the binding pocket.

Importantly, pyrazoles showing lower binding energy values correlated well with enhanced antimicrobial activity, as reflected by their MIC values. These findings highlight that strong and stable binding within the ATPase domain of DNA gyrase B is a key factor governing the antibacterial potential of the synthesized pyrazoles, as evidenced in Figure 2.

The pharmacokinetic properties of pyrazoles **4a–4h** were evaluated in silico, with particular emphasis on **lipophilicity (clog P)**, which is a key determinant of membrane permeability and oral bioavailability. The calculated log *p* values fall within an acceptable range for drug‐like molecules, indicating a balanced hydrophilic–lipophilic profile. The relatively higher lipophilicity observed for biphenyl‐ and polyaromatic‐substituted derivatives may facilitate bacterial membrane penetration, which is consistent with their enhanced antimicrobial activity. However, excessive lipophilicity could adversely affect solubility and systemic exposure, suggesting that fine tuning of aromatic substitution may further optimize the pharmacokinetic behavior of this scaffold. Overall, the predicted pharmacokinetic profiles support the drug‐likeness of **pyrazole 4h** (clog *p* = 7.3390) while highlighting lipophilicity as an important parameter governing their biological performance.

### 3.5 Statistical analysis

Statistical evaluation of the antimicrobial activity data confirmed that the synthesized compounds exhibited significant differences in their biological performance compared to the control drug Ciprofloxacin. Among the tested compounds, **pyrazole 4h** showed the highest activity, with statistically significant inhibition zones (*p* < 0.05) when compared to other derivatives.

The ANOVA results indicated a significant variation among the synthesized compounds, confirming that structural modifications had a measurable impact on biological activity. Post hoc analysis further revealed that compounds bearing electron‐withdrawing substituents demonstrated significantly enhanced activity compared to electron‐donating substituents.

Overall, the statistical analysis validated the reproducibility of the experimental data and supported the reliability of the observed SAR trends.

### 3.6 SAR investigation

Structure–activity relationship (SAR) analysis revealed that the antimicrobial potency of the synthesized pyrazole derivatives is strongly dependent on aromatic extension, heteroatom content, and lipophilicity. Among all derivatives, pyrazole 4h exhibited the highest antibacterial and antifungal activity along with the most favorable docking score (Δ*G* = −8.9 kcal/mol) against the FabB enzyme. The enhanced activity of pyrazole 4h may be attributed to its extended polyaromatic framework and multiple heteroatoms, which facilitate stable hydrogen bonding, hydrophobic interactions, and π–π stacking within the active site. In comparison, pyrazole 4d displayed moderate activity due to its partially extended aromatic system and comparatively lower heteroatom density. The less active derivatives lack sufficient aromatic surface area and suitable electronic features required for strong binding interactions. These findings suggest that balanced lipophilicity and extended π‐conjugation are important structural factors governing antimicrobial activity and provide valuable insights for the rational design of future pyrazole‐based antimicrobial agents.

## 4 CONCLUSION

The study reports the design and synthesis of a new series of pyrene‐integrated pyrazole derivatives, confirmed by FT‐IR, High‐resolution mass spectrometry, and NMR spectroscopy. Antimicrobial evaluation revealed a clear SAR, where substituent variation significantly influenced biological activity. Among all compounds, 4h exhibited the highest antibacterial and antifungal efficacy, supported by molecular docking results showing strong binding affinity toward *Escherichia coli* FabB (Δ*G* = −8.9 kcal/mol) through π‐conjugation–driven hydrophobic interactions and hydrogen bonding. It also showed notable inhibition zones against *Staphylococcus aureus* (38 mm) and *Proteus vulgaris* (31 mm), along with favorable drug‐likeness (cLogP = 7.3390). Overall, the results highlight 4h as a promising lead compound with potential socio‐economic relevance in the development of new antimicrobial agents against resistant pathogens, and suggest further optimization and mechanistic and in vivo studies for future advancement.

## AUTHOR CONTRIBUTIONS

All the experiments were designed and performed by Dinkal Kasundra and Paresh Patel. The spectral study, interpretation, and correlations were done by Dinkal Kasundra and Paresh Patel. The manuscript was written by Dinkal Kasundra. The final proofreading and editing were done by Paresh Patel.

## CONFLICT OF INTEREST STATEMENT

The authors declare no conflicts of interest.

## ETHICS STATEMENT

This study did not involve human participants or animal subjects requiring ethical approval.

## Supporting information

Supporting Information S1

## Data Availability

The data that support the findings of this study are available from the corresponding author upon reasonable request.
