# Supplementary material for: Pyrene‐linked heterocyclic pyrazoles: Synthesis, antimicrobial screening, and in silico molecular insights
Source: Smart Mol. 2026 Jul 28:e70074. Online ahead of print. doi: 10.1002/smo2.70074 (PMC13410603; doi:10.1002/smo2.70074)
Supplement: Supplementary file 1 — Supporting Information S1 [file SMO2-9999-0-s001.docx]

**Pyrene-Linked Heterocyclic Pyrazoles: Design, Synthesis, Antimicrobial Screening, and In Silico Molecular Insights**

*Dinkal V. Kasundra, Paresh N. Patel**

Laboratory of Bio-Organic Chemistry, Tarsadia Institute of Chemical Science (TICS),

Uka Tarsadia University, Bardoli – 394 350, Gujarat, India

e-mail:[pareshn111@yahoo.com](mailto:pareshn111@yahoo.com)*

**S1. Physical measurements**

All the chemicals were obtained from Sigma–Aldrich and were used without further purification. Solvents were dried over molecular sieves if necessary. The ^1^H NMR spectra were recorded in DMSO at room temperature using a Bruker AVANCE III 400 MHz (AV 400) multi nuclei solution NMR Spectrometer, TMS was used as internal reference, integration, multiplicity (s = singlet, d = doublet, t = triplet, q = quartet, quin = quintet, m = multiplate, br = broad, app = apparent), coupling constants (J; Hz), and assignment. ^13^C NMR spectra were measured on Bruker AVANCE III 100 MHz (AV 100) with complete proton decoupling. Chemical shifts were reported in ppm from the residual solvent as an internal standard. Infrared (IR) spectra were recorded neat by ATR on a Thermo Nicolet iS50 FT–IR spectrometer and are reported in cm^-1^. HR–MS data were obtained in methanol, with Thermo Scientific Orbi trap Elite Mass spectrometer. Melting point is measured by open capillary method using Sigma Melting Point Apparatus. Thin layer chromatography (TLC) analysis throughout this work, Merck percolated TLC plates (silica gel 60 GF254, 0.25 mm) were use. The products were purified by column chromatography recrystallization or column chromatography silica gel 60 (Merck, 230–400 mesh).

**S2. Spectral data**

**2-(1-Phenyl-3-(pyren-1-yl)-1*H*-pyrazol-5-yl)pyridine(4a):** Yellow solid; MP Above 300 °C; ^1^H NMR (400 MHz, DMSO) δ ppm: 8.173 (d, *J* = 8.3 Hz, 1H), 8.064 (t, *J* = 8.8 Hz, 1H), 7.955 (s, 1H), 7.865 (dd, *J* = 15.0, 8.3 Hz, 2H), 7.783 (dd, *J* = 12.3, 5.0 Hz, 2H), 7.612 (s, 1H), 7.548-7.449 (m, 2H), 7.155 (d, *J* = 3.4 Hz, 1H), 6.725 (dd, *J* = 3.4, 1.7 Hz, 1H); ^13^C NMR (100 MHz, CDCl_3_) δ ppm: 142.97, 136.05, 133.64, 133.25, 131.33, 131.19, 130.72, 129.79, 129.73, 129.60, 129.47, 129.42, 128.98, 128.71, 127.24, 126.64, 126.56, 126.50, 126.31, 126.14, 125.47, 125.25, 124.90, 124.17; FT-IR (Bands cm^-1^): 1630, 1300, 1284, 1104; HRMS (ESI) [M+H]^+1^: calculated for C_30_H_19_N_3_: 422.1579, found: 422.1559.

**3-(1-Phenyl-3-(pyren-1-yl)-1*H*-pyrazol-5-yl)pyridine(4b):** Yellow solid; MP Above 300 °C; ^1^H NMR (400 MHz, DMSO) δ ppm: 8.734 (d, *J* = 3.3 Hz, 1H), 8.244 – 8.205 (m, 4H), 7.955 – 7.884 (m, 5H), 7.814 – 7.765 (m, 4H), 7.565 – 7.505 (m, 2H), 7.495 – 7.436 (m, 3H); ^13^C NMR (100 MHz, CDCl_3_) δ ppm: 145.66, 143.50, 140.08, 133.67, 131.19, 130.73, 129.22, 129.13, 129.07, 128.95, 127.97, 127.65, 127.37, 127.24, 127.07, 126.43, 126.25, 126.14, 126.0 0, 125.47, 124.96, 124.76, 124.13; FT-IR (Bands cm^-1^): 1630, 1300, 1284, 1104; HRMS (ESI) [M+H]: calculated for C_30_H_19_N_3_: 421.1579, found: 421.1565.

**4-(1-Phenyl-3-(pyren-1-yl)-1*H*-pyrazol-5-yl) pyridine(4c)**: Yellow solid; MP Above 300 °C; ^1^H NMR (400 MHz, CDCl_3_) δ ppm: 8.165 – 8.105 (m, 1H), 8.046 (d, *J* = 6.7 Hz, 1H), 7.937 (s, 1H), 7.886 (dd, *J* = 15.7, 3.2 Hz, 1H), 7.785 – 7.585 (m, 9H), 7.525 – 7.405 (m, 6H); ^13^C NMR (100 MHz, DMSO) δ ppm: 151.23, 147.72, 144.77, 142.96, 140.65, 140.17, 139.87, 136.13, 132.30, 131.82, 131.30, 130.77, 129.48, 129.35, 128.94, 127.00, 125.24, 124.47, 123.16, 119.23, 113.42, 108.53; FT-IR (Bands cm^-1^): 1630, 1300, 1284, 1104; HRMS (ESI) [M+H]: calculated for C_30_H_19_N_3_: 421.1579, found: 421.1564.

**1-Phenyl-3-(pyren-1-yl)-5-(thiophen-2-yl)-1*H*-pyrazole(4d):** Light brown solid; MP Above 300 °C; ^1^H NMR (400 MHz, CDCl_3_) δ ppm: 8.165 – 8.103 (m, 1H), 8.045 (d, *J* = 6.7 Hz, 1H), 7.935 (s, 1H), 7.883 (dd, *J* = 15.7, 3.2 Hz, 1H), 7.784 – 7.595 (m, 4H), 7.535 – 7.395 (m, 3H); ^13^C NMR (100 MHz, DMSO) δ ppm: 148.27, 146.67, 144.79, 140.92, 140.33, 139.57, 139.16, 137.64, 133.13, 131.13, 129.76, 129.51, 128.95, 127.53, 127.39, 127.07, 126.94, 125.65, 125.23, 124.92, 124.09, 123.47, 123.13, 122.13, 119.77, 114.01; FT-IR (Bands cm^-1^): 1630, 1300, 1284, 1104; HRMS (ESI) [M+H]^+1^: calculated for C_29_H_18_N_2_S: 427.1191, found: 427.1177.

**5-(Furan-2-yl)-1-phenyl-3-(pyren-1-yl)-1*H*-pyrazole(4e):** Yellow solid; MP Above 300 °C; ^1^H NMR (400 MHz, CDCl_3_) δ ppm: 8.123 (td, *J* = 4, 4 Hz, 2H), 8.064 (dd, *J* = 15.2, 0.5 Hz, 2H), 7.855 – 7.783 (m, 2H), 7.775 – 7.736 (dt, *J* = 4, 3.4 Hz, 2H), 7.686 – 7.645 (m, 2H), 7.593 (s, 1H), 7.523 – 7.456 (m, 4H), 7.445 – 7.376 (m, 3H); ^13^C NMR (100 MHz, CDCl_3_) δ ppm: 137.43, 134.05, 133.36, 132.13, 131.96, 131.19, 130.71, 130.53, 129.78, 129.68, 129.23, 126.13, 125.21, 125.11, 125.00, 124.76, 124.15, 124.01, 123.10, 122.20, 112.53; FT-IR (Bands cm^-1^): 1630, 1300, 1284, 1104; HRMS (ESI) [M+H]^+1^: calculated for C_29_H_18_N_2_O: 411.1419, found: 411.1409.

**5-(Benzo[*b*]thiophen-2-yl)-1-phenyl-3-(pyren-1-yl)-1*H*-pyrazole(4f):** Yellow solid; MP Above 300 °C; ^1^H NMR (400 MHz, DMSO) δ ppm: 8.325 (t, *J* = 7.0 Hz, 1H), 8.105 (t, *J* = 8.0 Hz, 1H), 8.023 (dd, *J* = 14.1, 7.6 Hz, 2H), 7.866 – 7.715 (m, 5H), 7.595 – 7.456 (m, 6H), 7.407 (t, *J* = 7.3 Hz, 1H), 7.323 – 7.165 (m, 4H); ^13^C NMR (100 MHz, DMSO) δ ppm: 148.15, 145.00, 141.19, 140.72, 139.90, 136.78, 136.57, 131.73, 129.75, 129.50, 128.19, 127.53, 127.36, 127.04, 126.84, 125.14, 124.90, 124.45, 123.82, 122.32, 119.38, 113.55; FT-IR (Bands cm^-1^): 1630, 1300, 1284, 1104; HRMS (ESI) [M+H]^+1^: calculated for C_33_H_20_N_2_S: 476.1347, found: 476.1334.

**5-(Benzo[*b*]thiophen-3-yl)-1-phenyl-3-(pyren-1-yl)-1*H*-pyrazole (4g):** Yellow solid; MP Above 300 °C; ^1^H NMR (400 MHz, CDCl_3_) δ ppm: 8.678 (d, *J* = 9.3 Hz, 1H), 8.335 – 8.174 (m, 8H), 8.155 – 8.055 (m, 3H), 7.896 (d, *J* = 15.5 Hz, 1H), 7.795 (dd, *J* = 20.3, 7.2 Hz, 2H), 7.515 (s, 1H), 7.435 – 7.314 (m, 4H); ^13^C NMR (100 MHz, DMSO) δ ppm: 148.15, 145.00, 141.19, 140.72, 139.90, 136.78, 136.57, 131.73, 129.75, 129.61, 129.50, 129.39, 128.19, 127.53, 127.36, 127.04, 126.84, 125.14, 124.90, 124.45, 123.82, 122.32, 120.39, 119.38, 113.55, 112.03; FT-IR (Bands cm^-1^): 1630, 1300, 1284, 1104; HRMS (ESI) [M+H]^+1^: calculated for C_33_H_20_N_2_S: 476.1347, found: 476.1324

**5-(1*H*-Imidazol-2-yl)-1-phenyl-3-(pyren-1-yl)-1*H*-pyrazole (4h):** Yellow solid; MP Above 300 °C; ^1^H NMR (500 MHz, DMSO) δ ppm: 8.625 (d, *J* = 15.4 Hz, 1H), 8.326 (dt, *J* = 6.7, 3.4 Hz, 4H), 8.134 – 8.026 (m, 3H), 7.927 (d, *J* = 8.4 Hz, 2H), 7.835 – 7.794 (m, 2H), 7.713 – 7.612 (m, 3H), 7.556 (dd, *J* = 10.4, 4.8 Hz, 2H), 7.465 (dd, *J* = 8.3, 6.4 Hz, 1H); ^13^C NMR (125 MHz, DMSO) δ ppm: 151.11, 143.92, 140.08, 139.50, 135.57, 132.09, 130.72, 129.70, 129.60, 129.47, 128.86, 128.44, 128.05, 127.53, 127.02, 126.36, 125.93, 125.86, 122.90, 105.97; FT-IR (Bands cm^-1^): 1630, 1300, 1284, 1104; HRMS (ESI) [M+H]^+1^: calculated for C_28_H_18_N_4_: 410.1531, found: 410.1510.

***5.6.2.2 Spectrum of prepared pyrazoles derivatives***


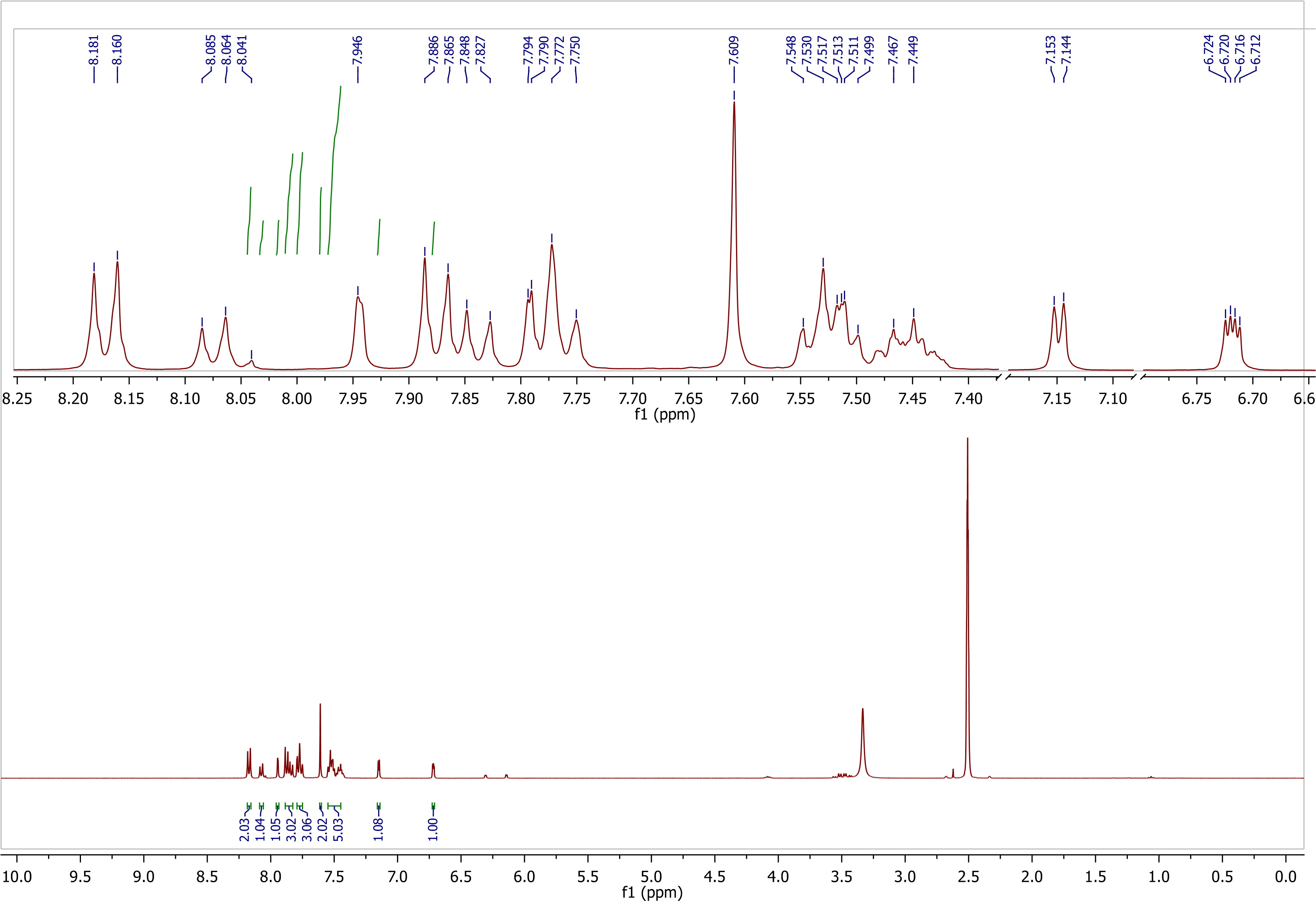


**Figure S1.** ^1^H NMR of 2-(1-Phenyl-3-(pyren-1-yl)-1*H*-pyrazol-5-yl) pyridine **(4a)**

***
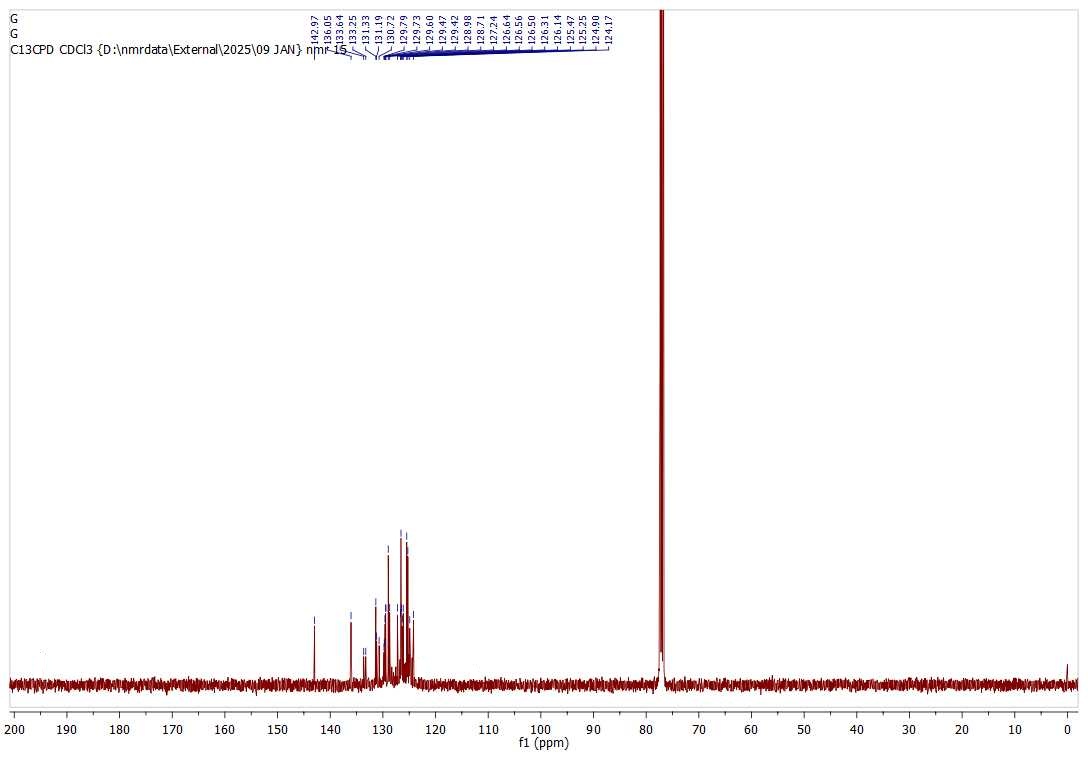
***

**Figure S2.** ^13^C NMR of 2-(1-Phenyl-3-(pyren-1-yl)-1*H*-pyrazol-5-yl) pyridine **(4a)**


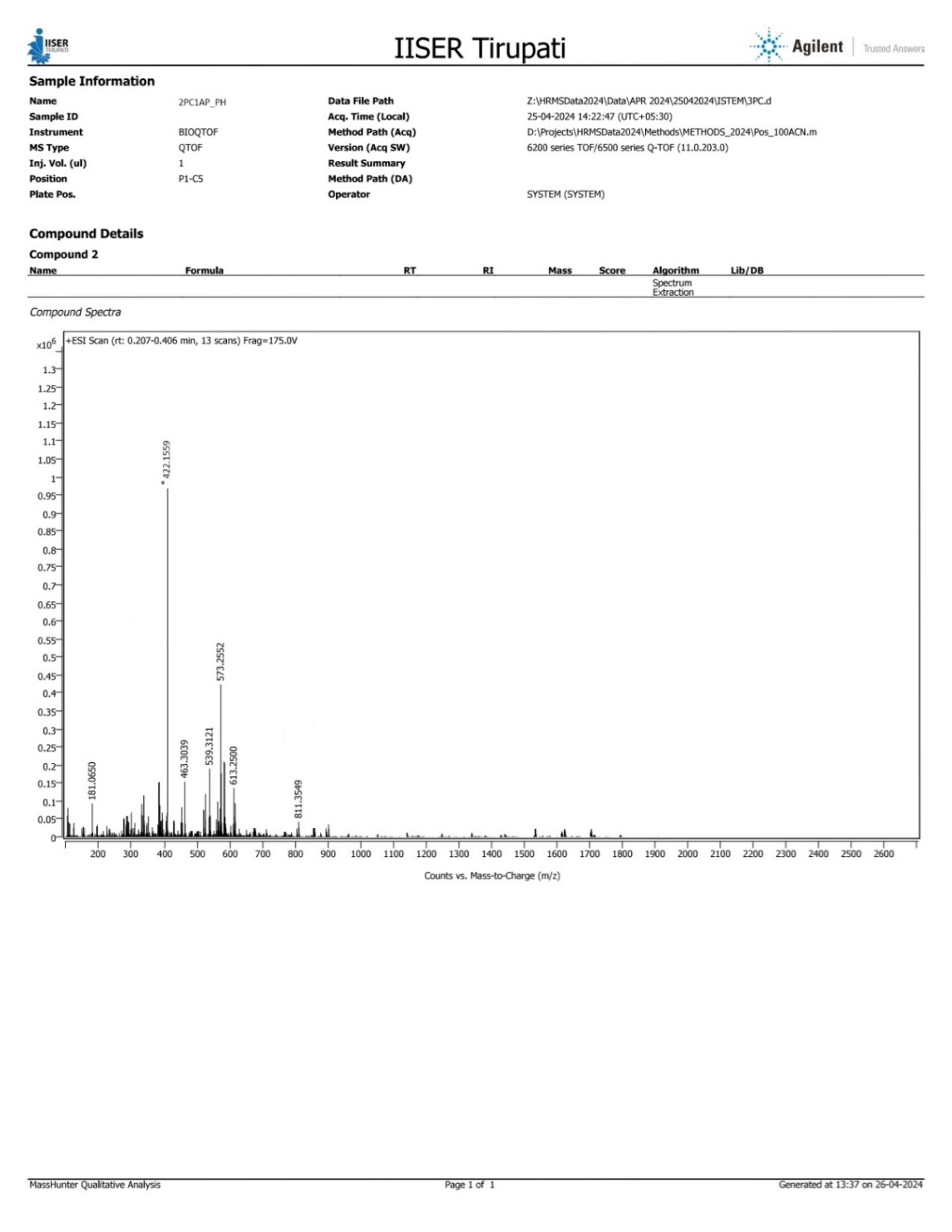


**Figure S3.** HRMS of 2-(1-Phenyl-3-(pyren-1-yl)-1*H*-pyrazol-5-yl) pyridine **(4a)**


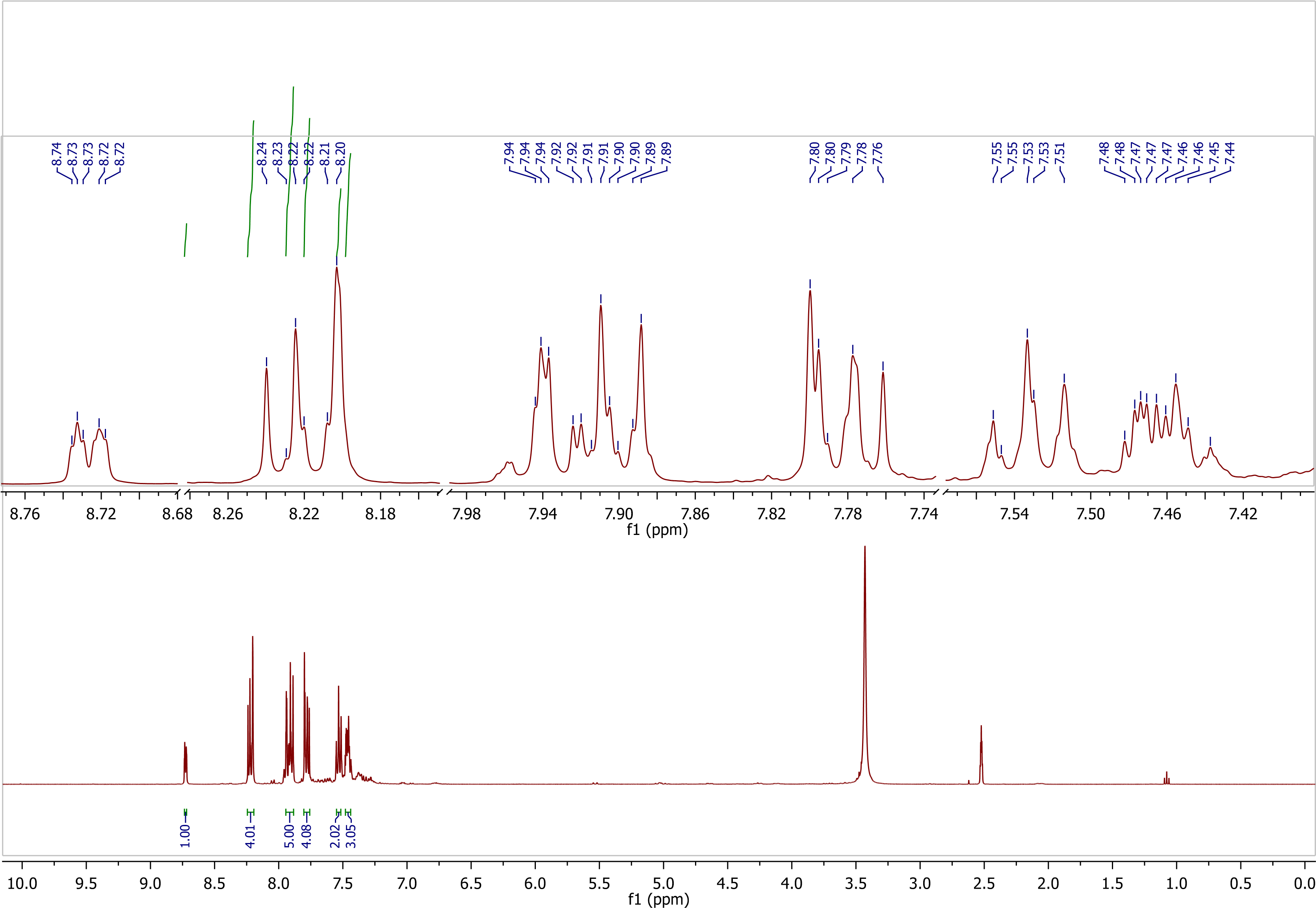


**Figure S4.** ^1^H NMR of 3-(1-Phenyl-3-(pyren-1-yl)-1*H*-pyrazol-5-yl) pyridine **(4b)**


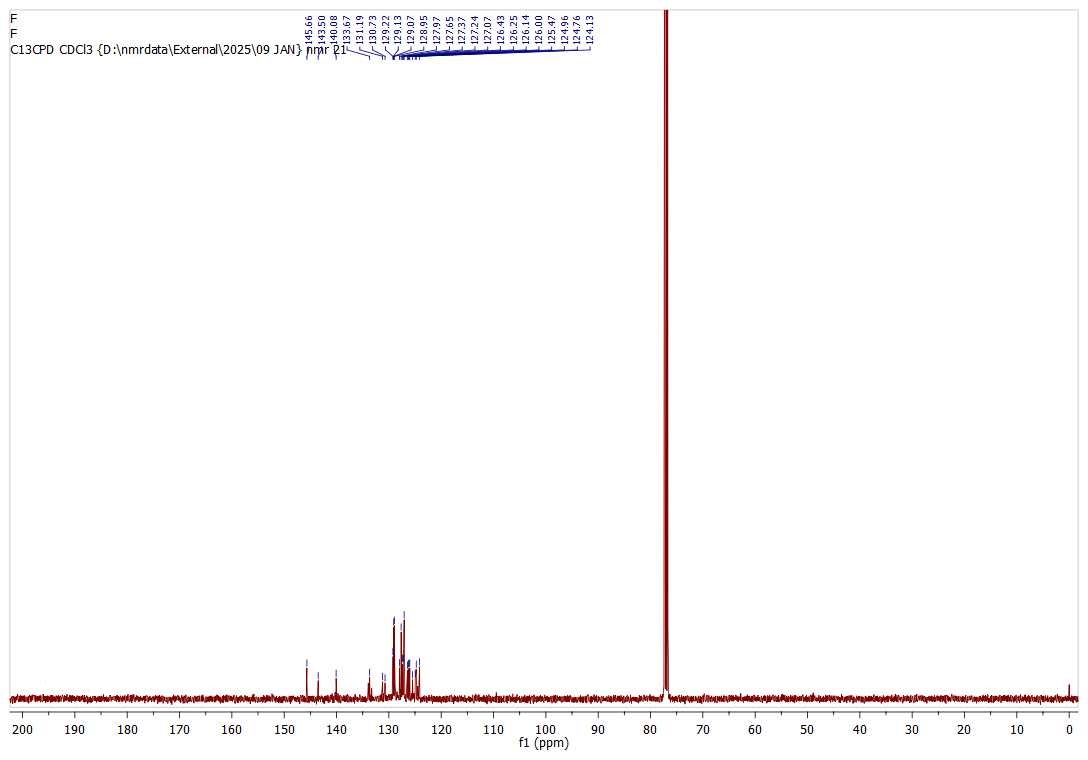


**Figure S5.** ^13^C NMR of 3-(1-Phenyl-3-(pyren-1-yl)-1*H*-pyrazol-5-yl) pyridine **(4b)**


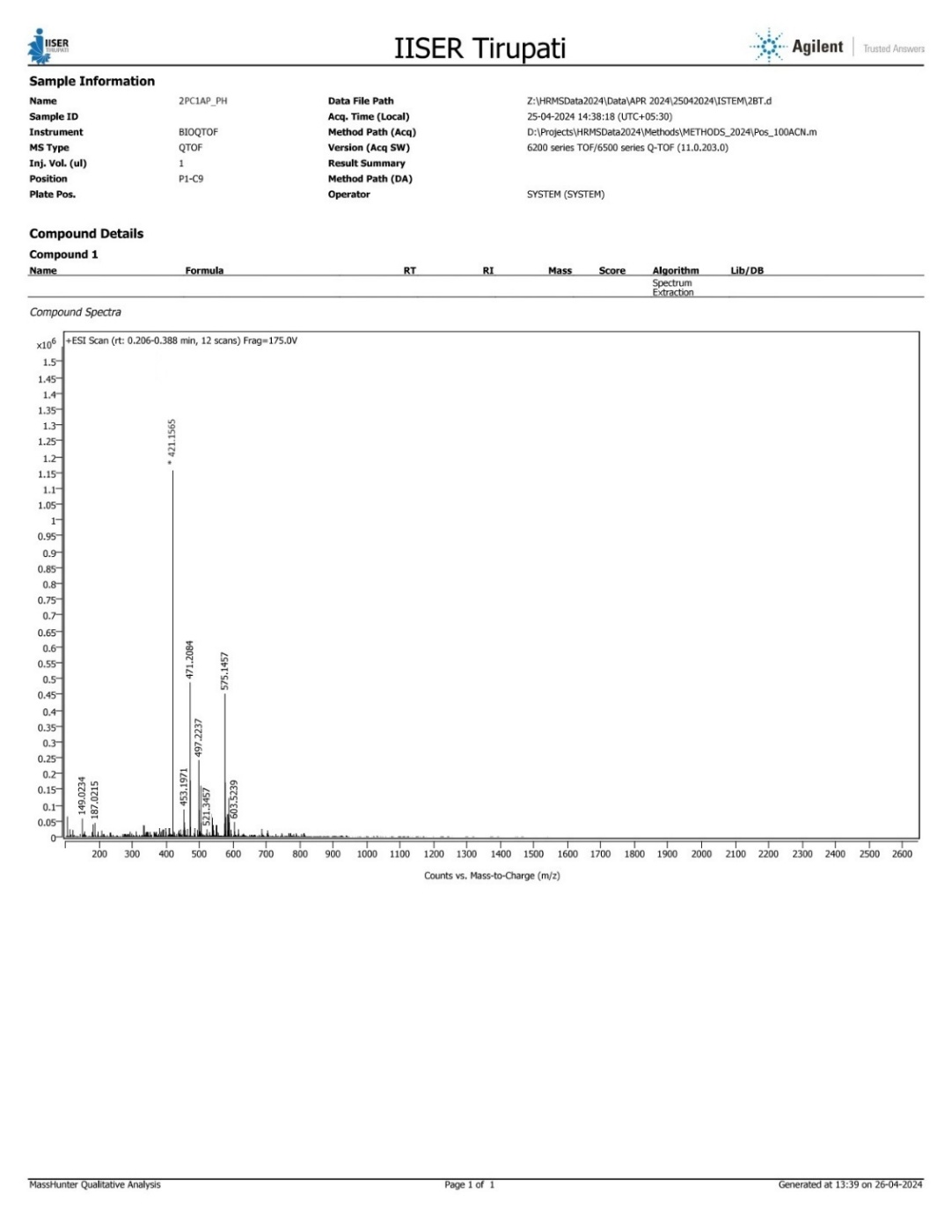


**Figure S6.**  HRMS of 3-(1-Phenyl-3-(pyren-1-yl)-1*H*-pyrazol-5-yl) pyridine **(4b)**


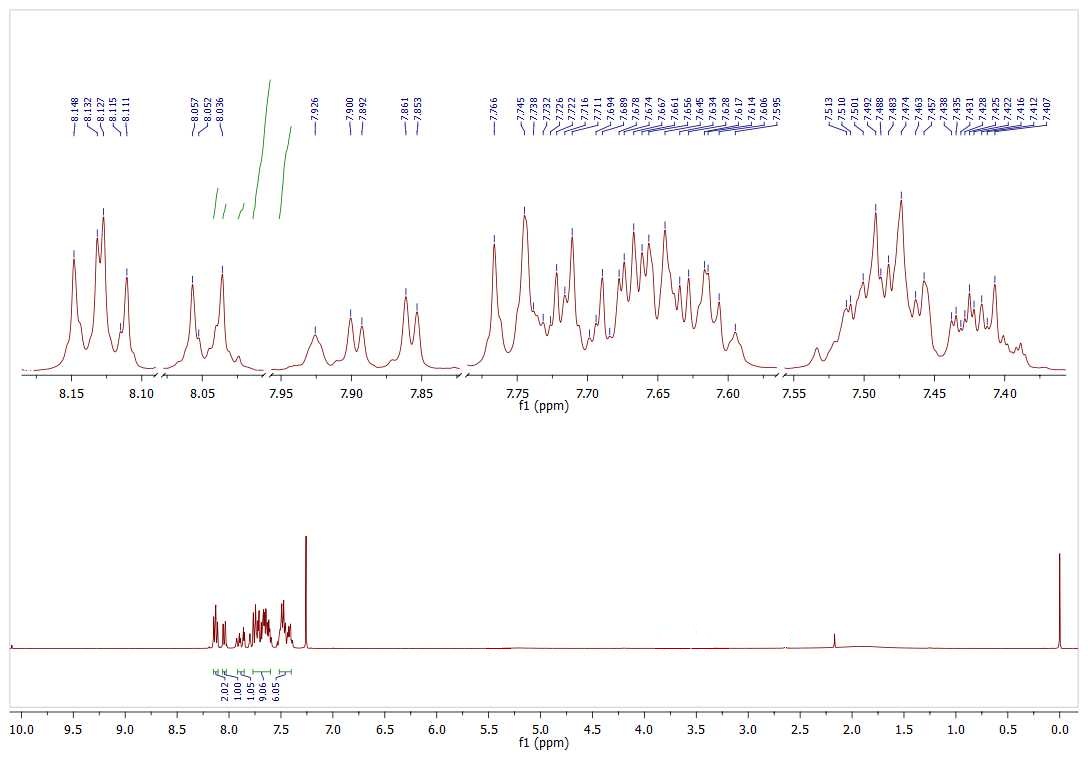


**Figure S7.** ^1^H NMR of 4-(1-Phenyl-3-(pyren-1-yl)-1*H*-pyrazol-5-yl) pyridine **(4c)**


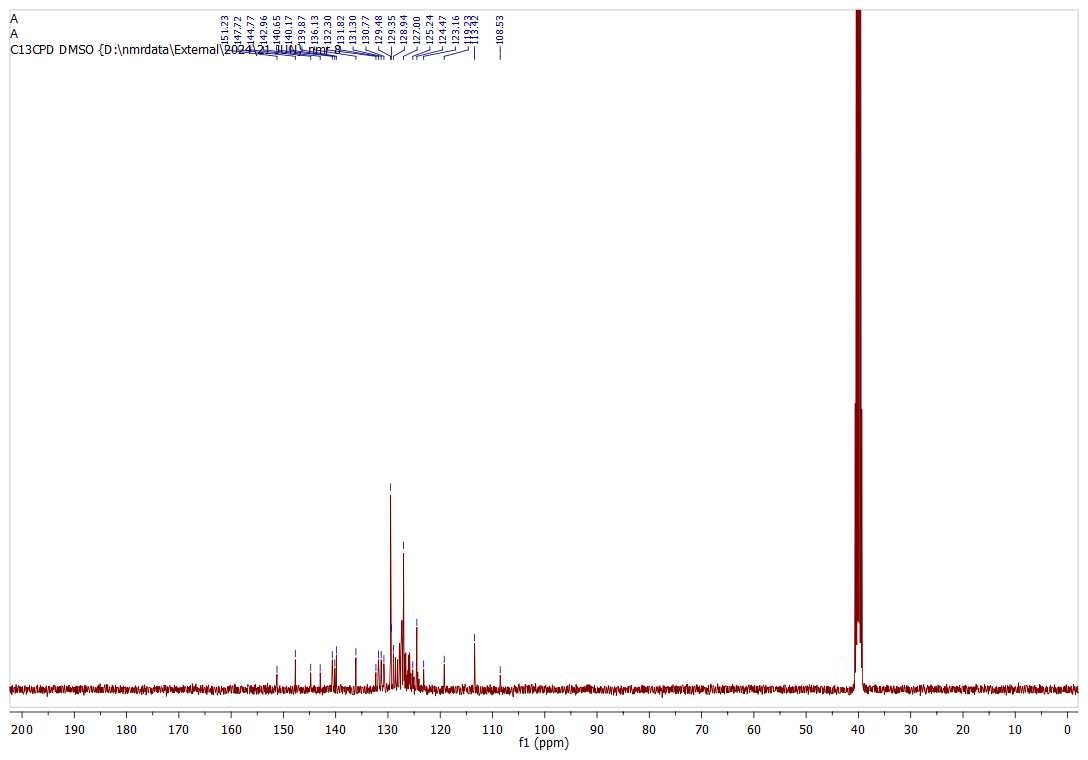


**Figure S8.** ^13^C NMR of 4-(1-Phenyl-3-(pyren-1-yl)-1*H*-pyrazol-5-yl) pyridine **(4c)**

**
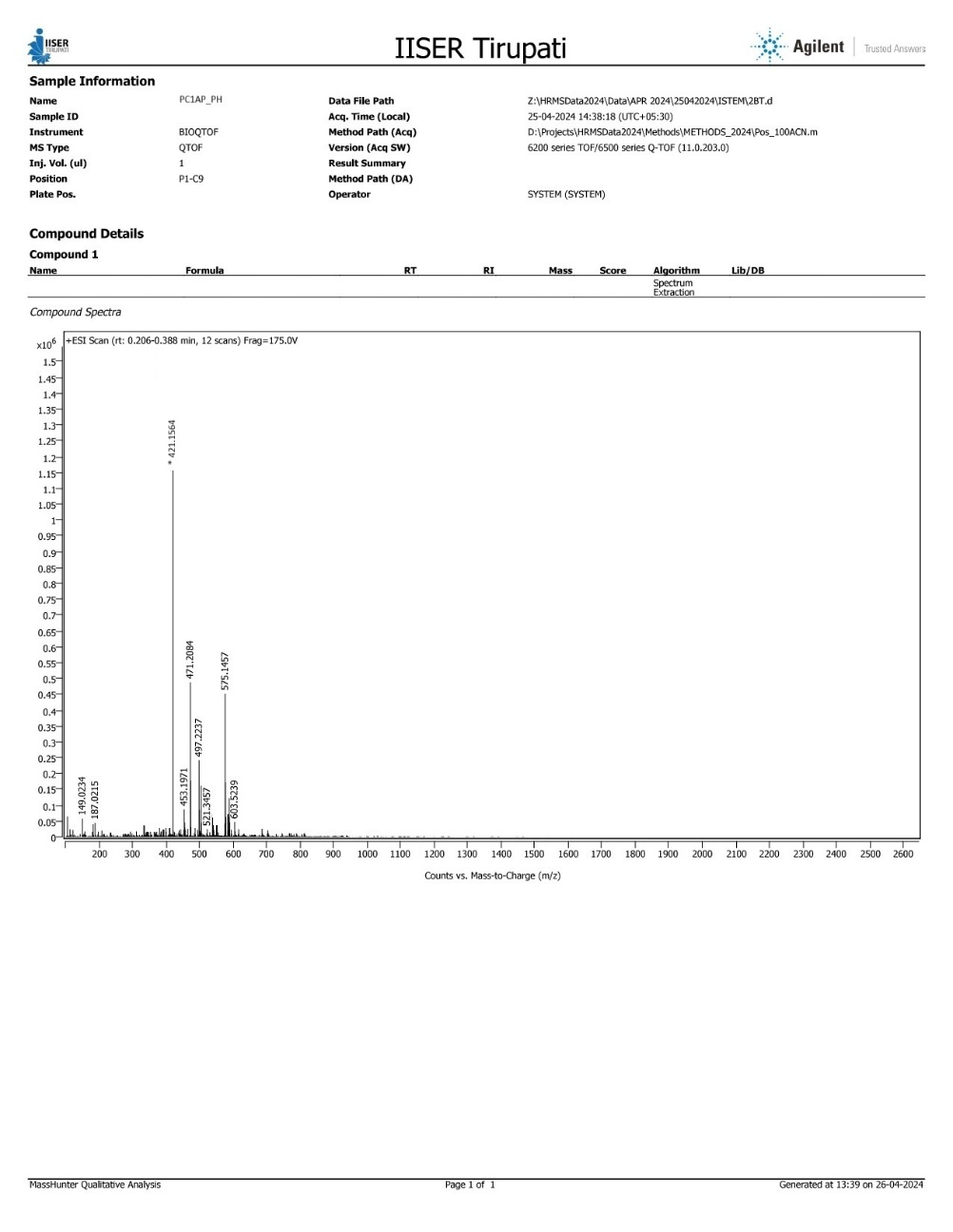
**

**Figure S9.** HRMS of 4-(1-Phenyl-3-(pyren-1-yl)-1*H*-pyrazol-5-yl) pyridine **(4c)**


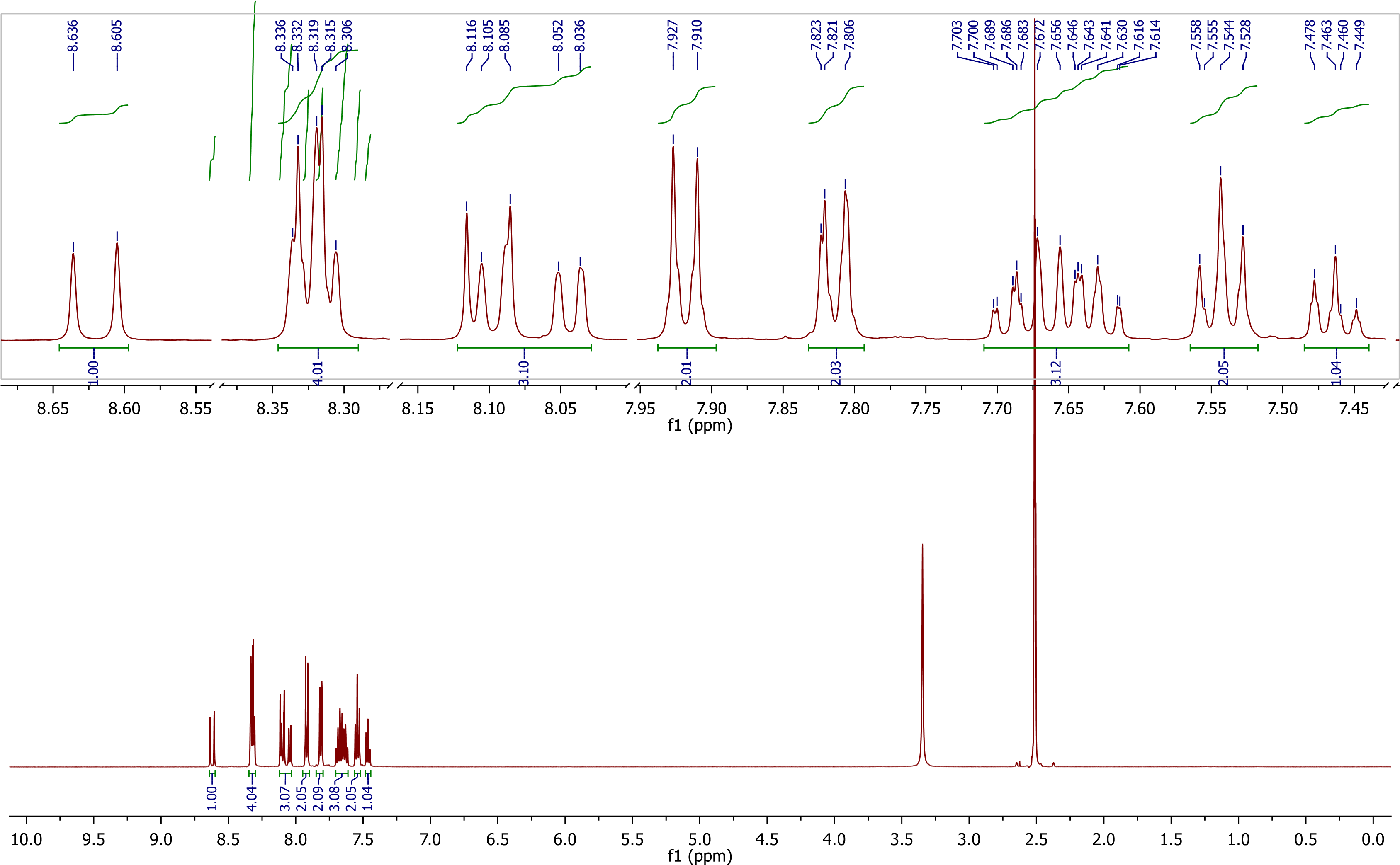


**Figure S10.** ^1^H NMR of 1-Phenyl-3-(pyren-1-yl)-5-(thiophen-2-yl)-1*H*-pyrazole **(4d)**


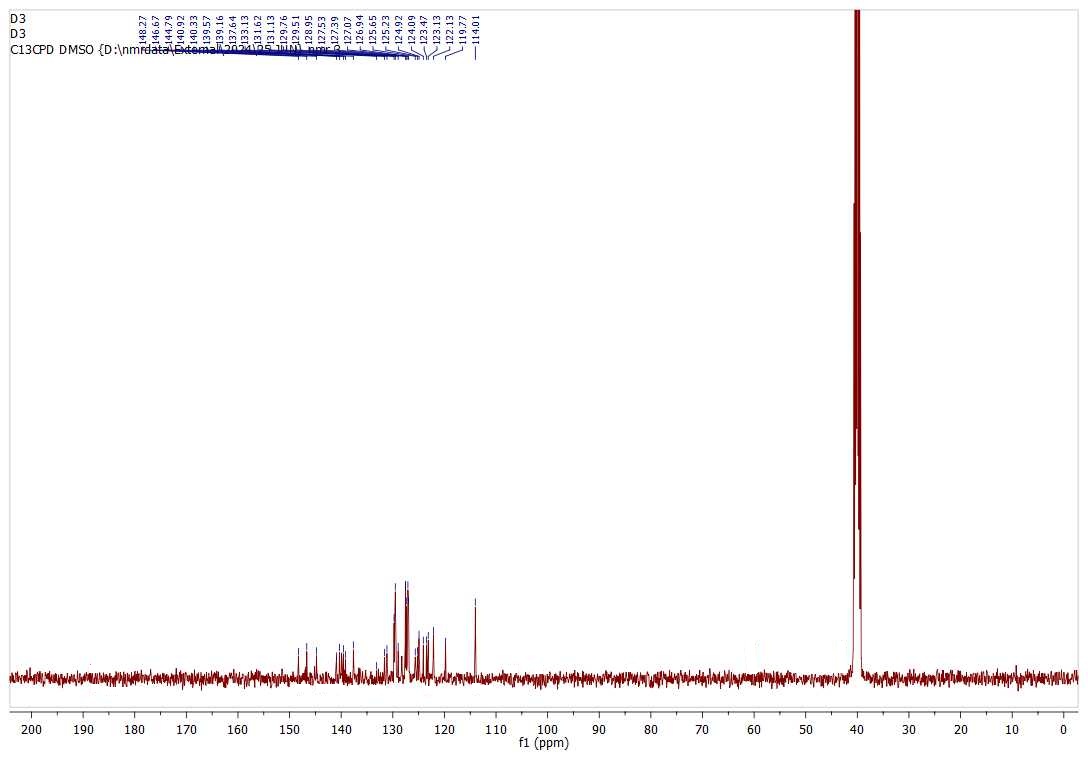


**Figure S11.** ^13^C NMR of 1-Phenyl-3-(pyren-1-yl)-5-(thiophen-2-yl)-1*H*-pyrazole **(4d)**


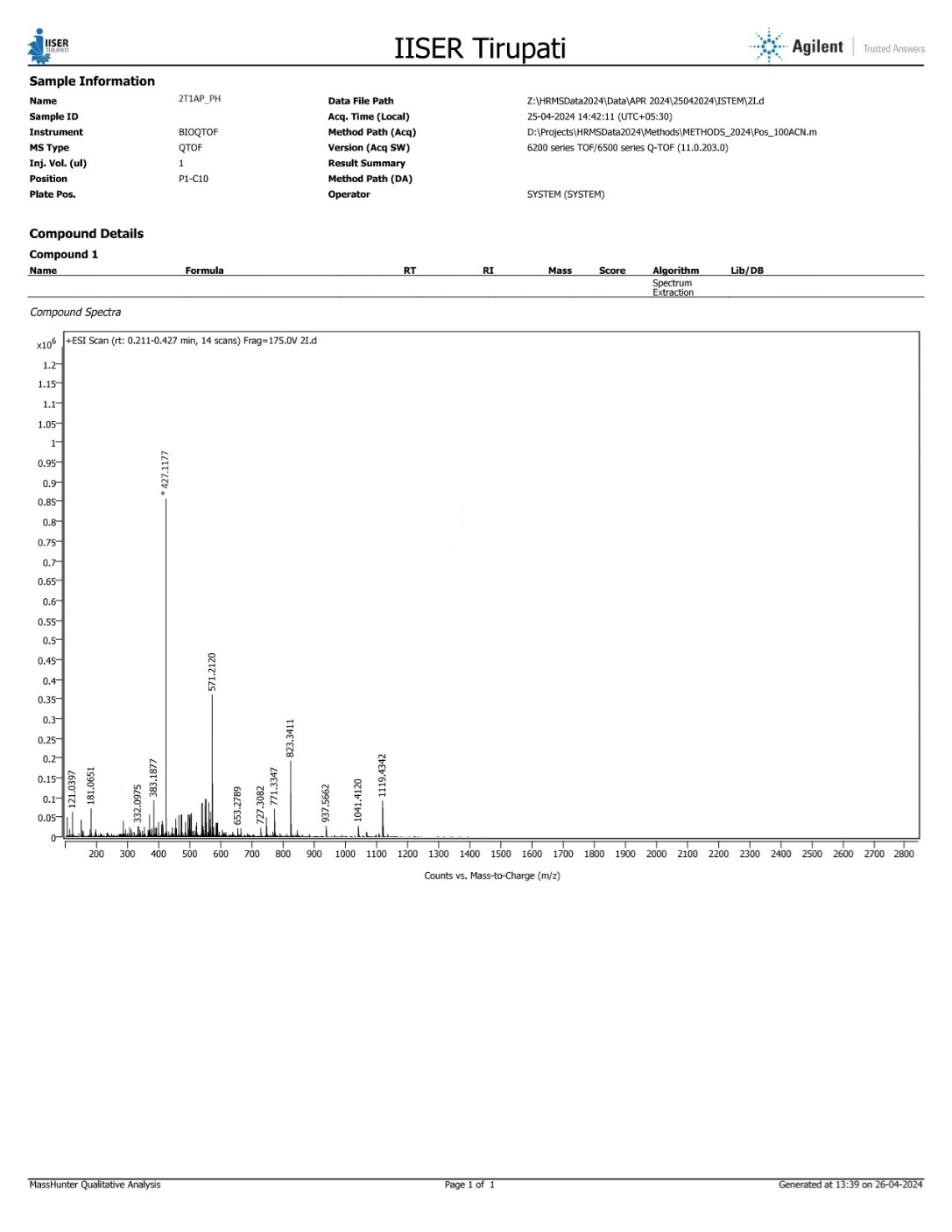


**Figure S12.** HRMS of 1-Phenyl-3-(pyren-1-yl)-5-(thiophen-2-yl)-1*H*-pyrazole **(4d)**


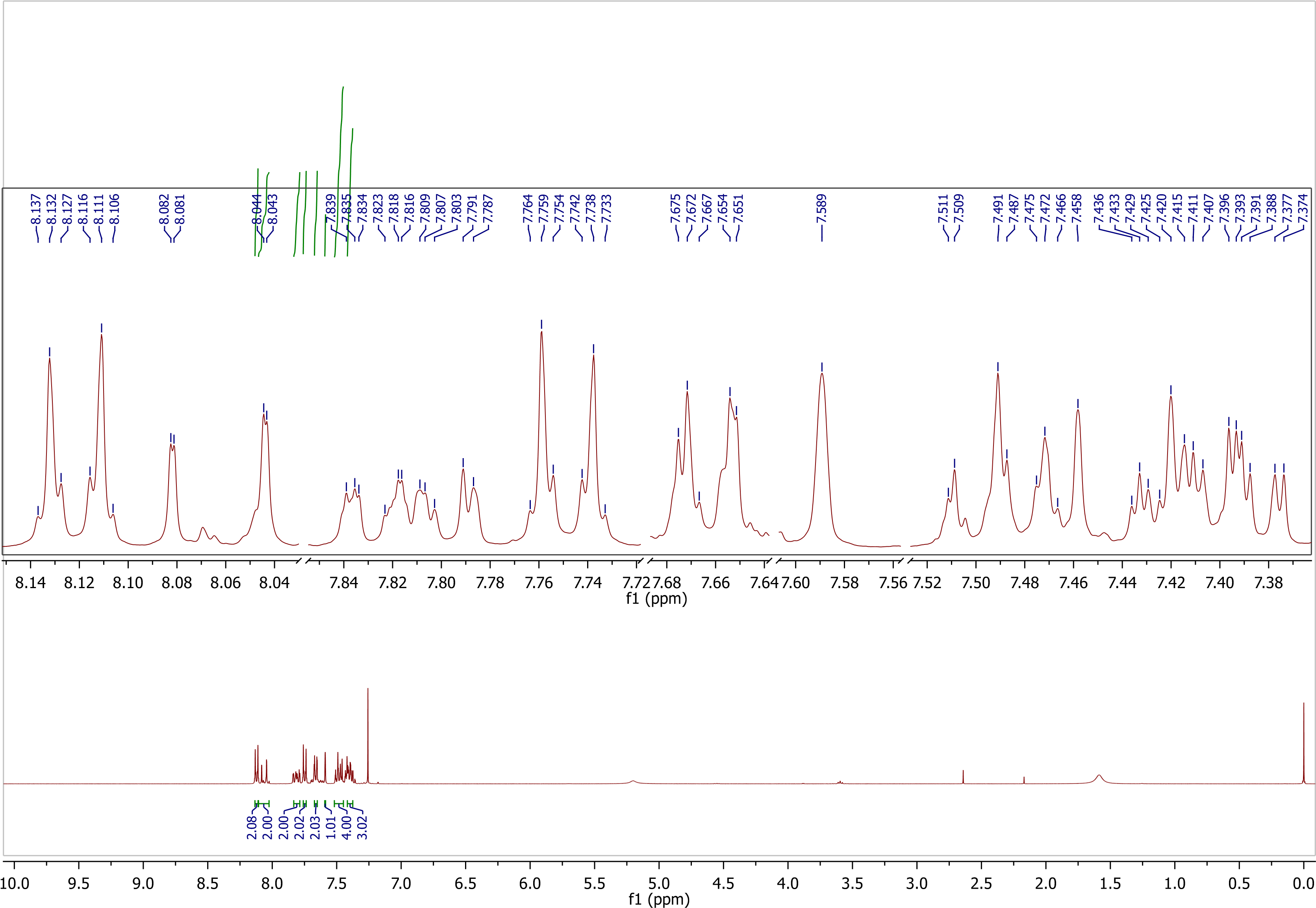


**Figure S13.** ^1^H NMR of 5-(Furan-2-yl)-1-phenyl-3-(pyren-1-yl)-1*H*-pyrazole **(4e)**


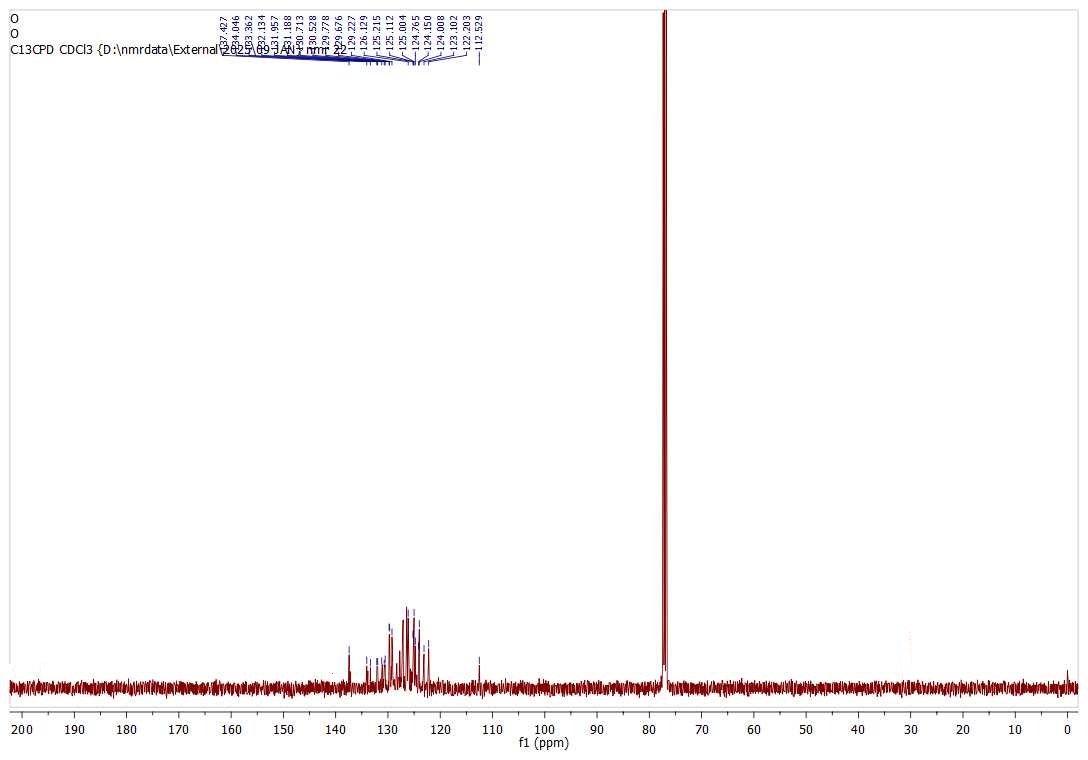


**Figure S14.**  ^13^C NMR of 5-(Furan-2-yl)-1-phenyl-3-(pyren-1-yl)-1*H*-pyrazole **(4e)**


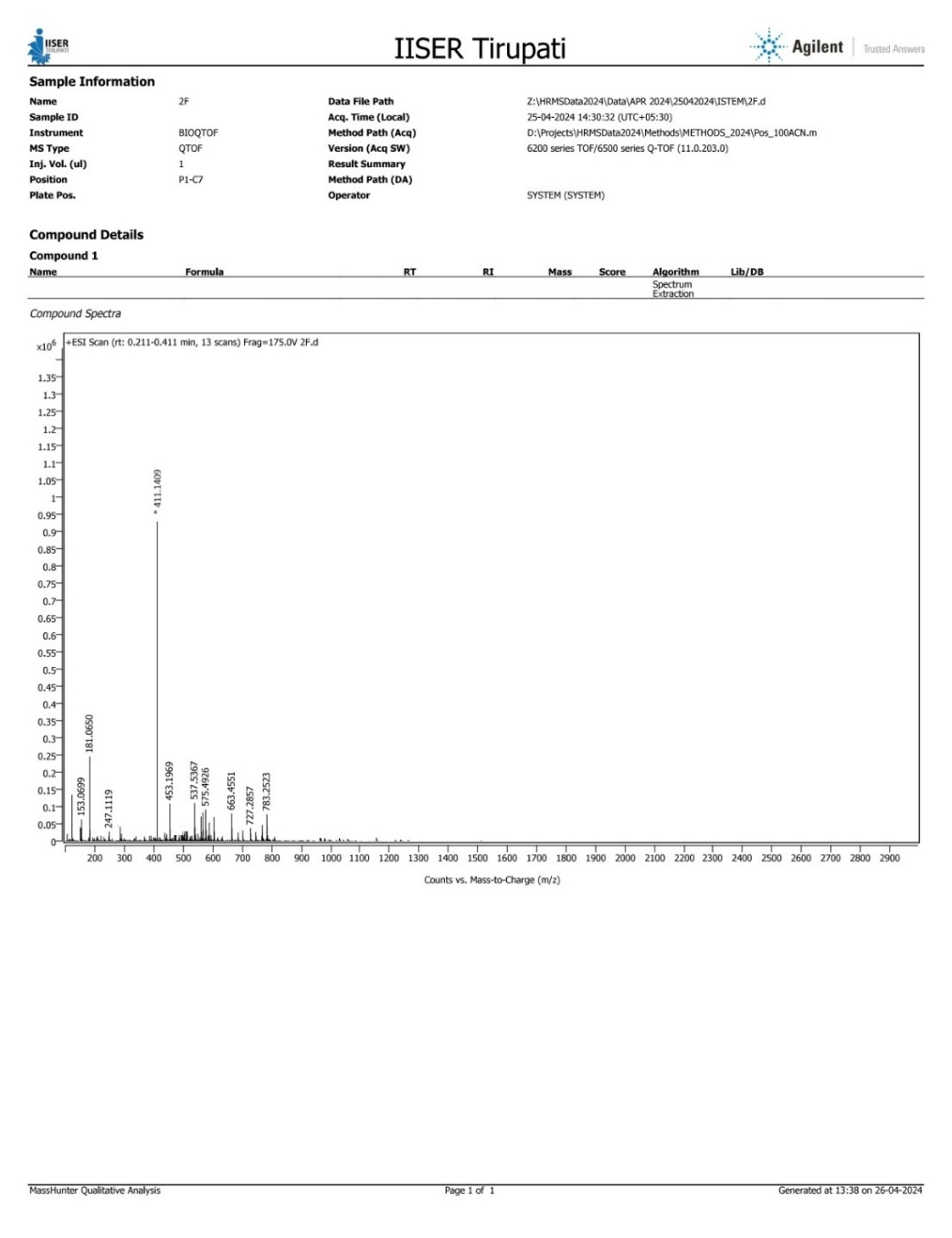


**Figure S15.**  HRMS of 5-(Furan-2-yl)-1-phenyl-3-(pyren-1-yl)-1*H*-pyrazole **(4e)**


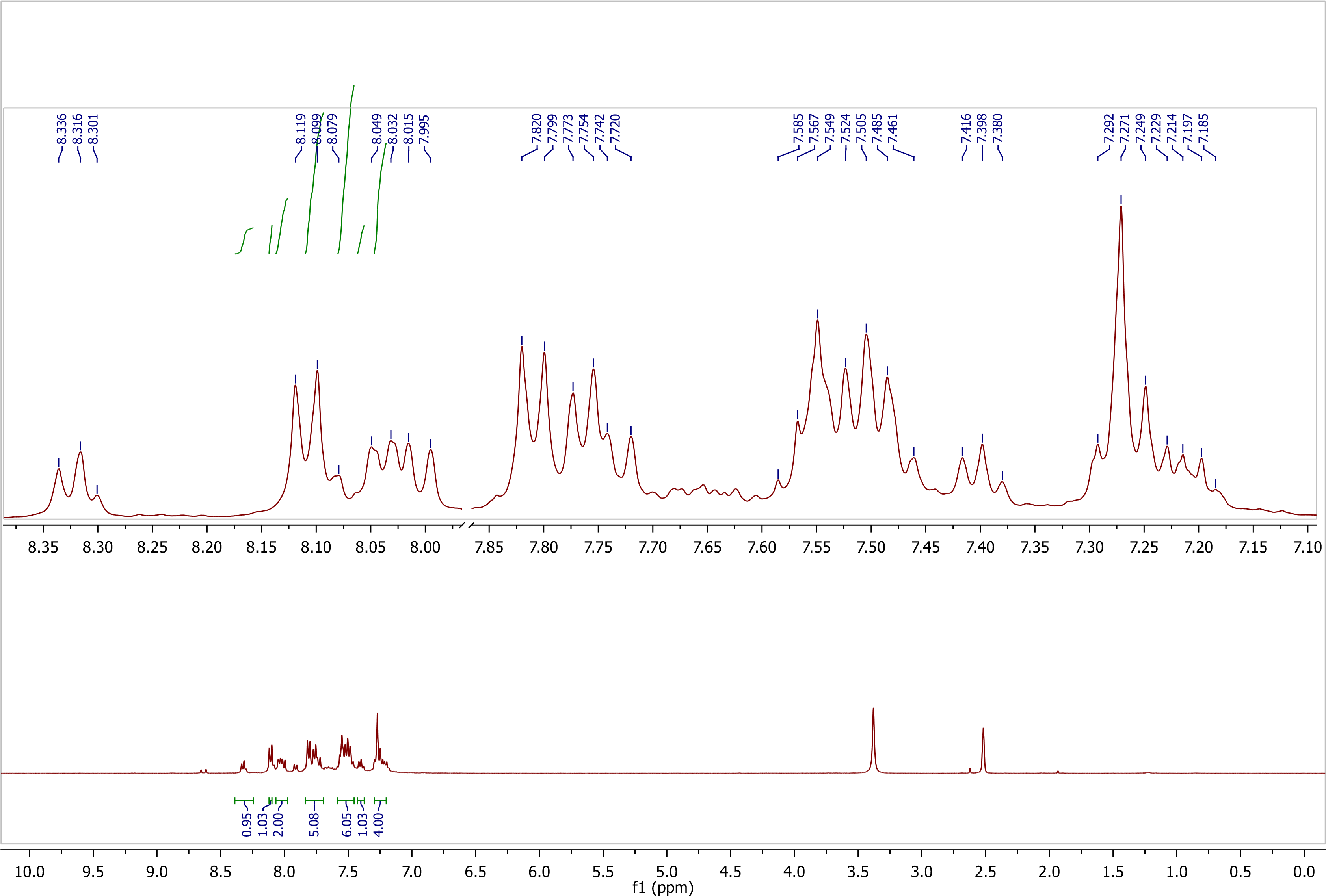


**Figure S16.** ^1^H NMR of 5-(Benzo[*b*]thiophen-2-yl)-1-phenyl-3-(pyren-1-yl)-1*H*-pyrazole **(4f)**


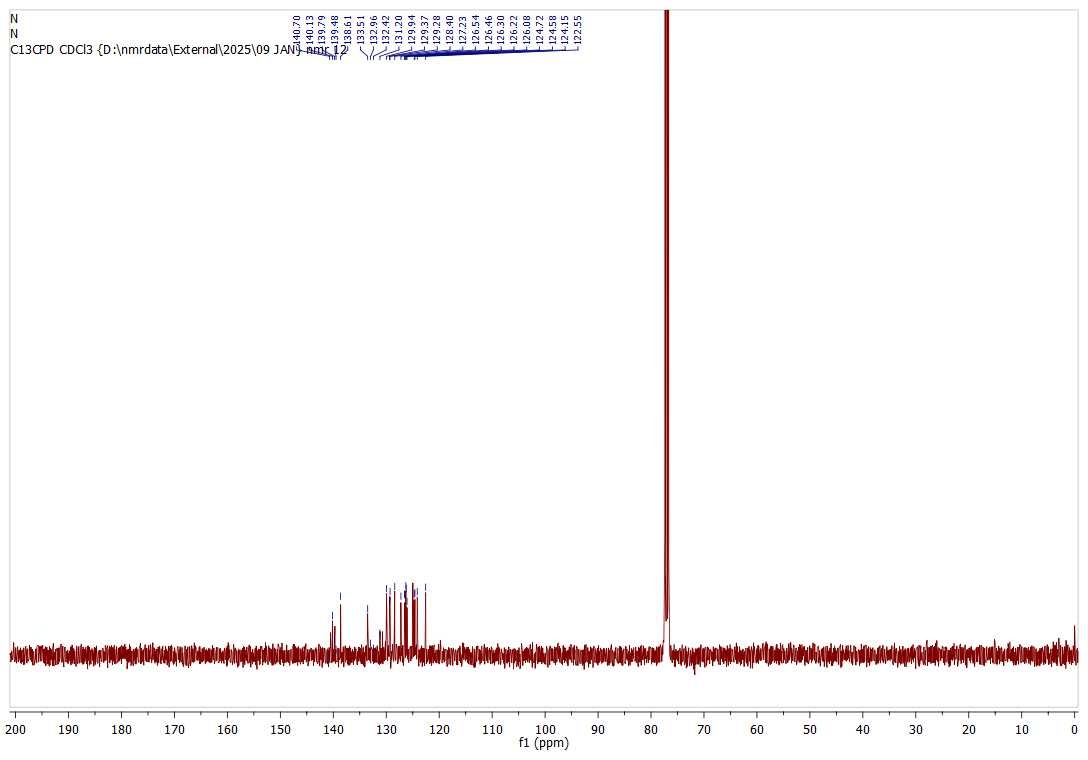


**Figure S17.** ^13^C NMR of 5-(Benzo[*b*]thiophen-2-yl)-1-phenyl-3-(pyren-1-yl)-1*H*-pyrazole **(4f)**


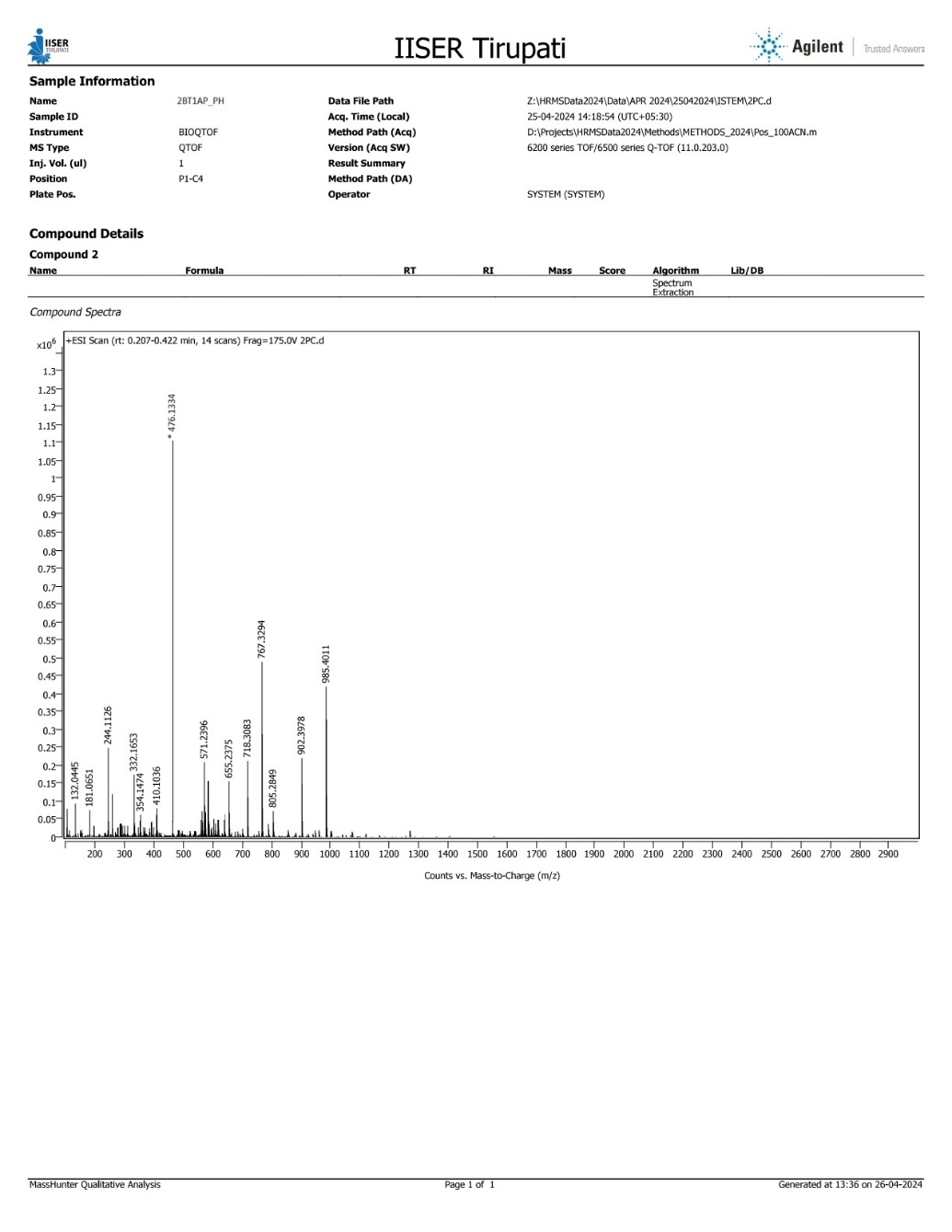


**Figure S18.**  HRMS of 5-(Benzo[*b*]thiophen-2-yl)-1-phenyl-3-(pyren-1-yl)-1*H*-pyrazole **(4f)**


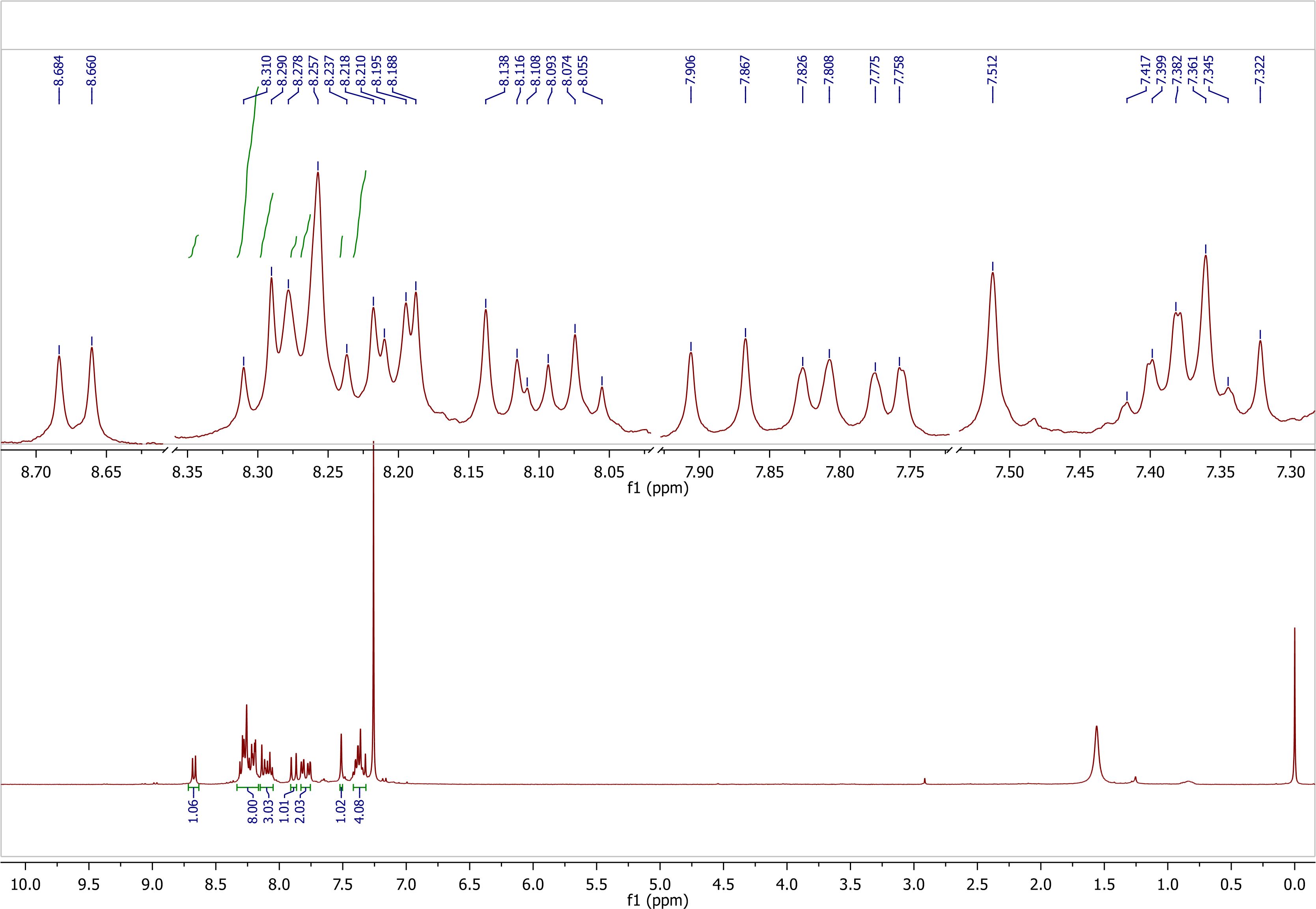


**Figure S19.** ^1^H NMR of 5-(Benzo[*b*]thiophen-3-yl)-1-phenyl-3-(pyren-1-yl)-1*H*-pyrazole **(4g)**


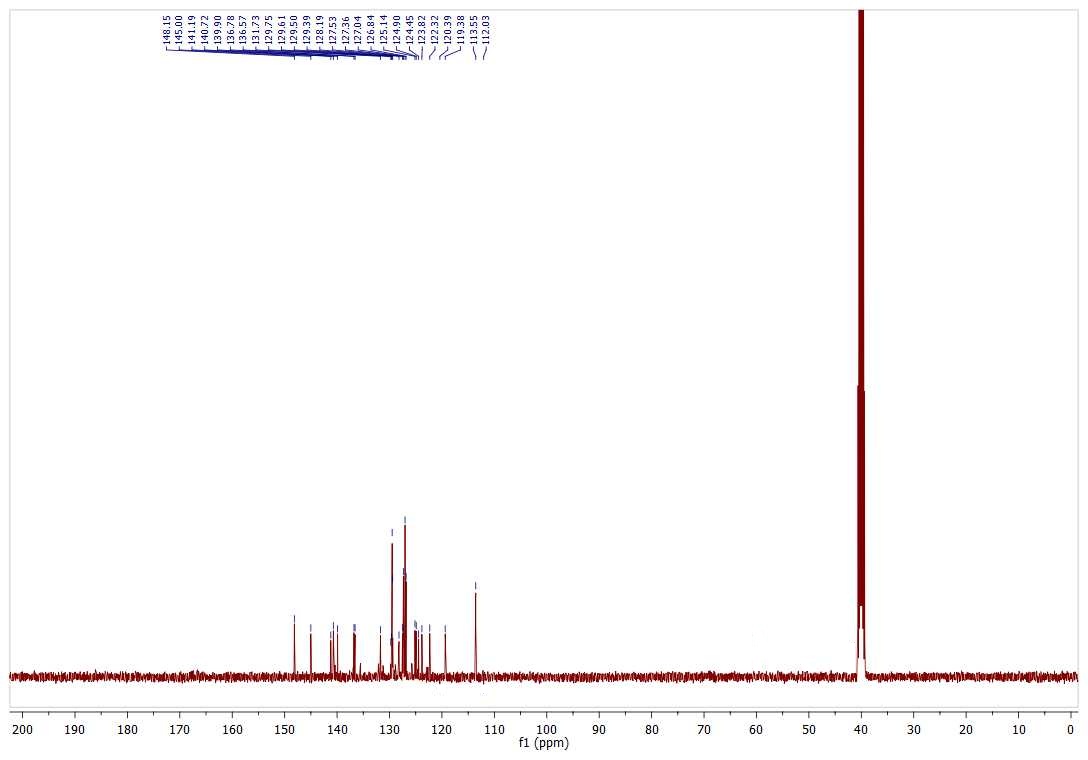


**Figure S20.** ^13^C NMR of 5-(Benzo[*b*]thiophen-3-yl)-1-phenyl-3-(pyren-1-yl)-1*H*-pyrazole **(4g)**


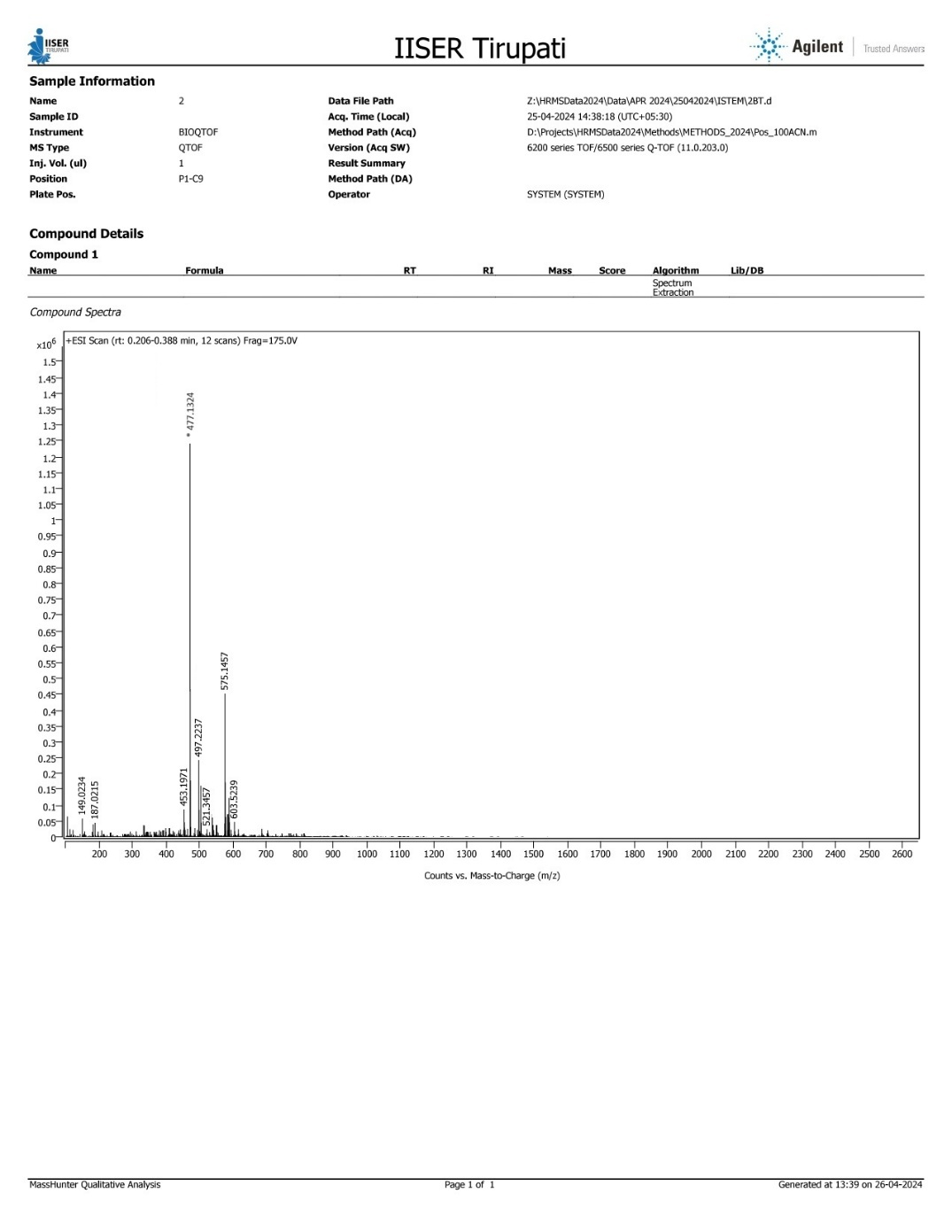


**Figure S21.** HRMS of 5-(Benzo[*b*]thiophen-3-yl)-1-phenyl-3-(pyren-1-yl)-1*H*-pyrazole **(4g)**


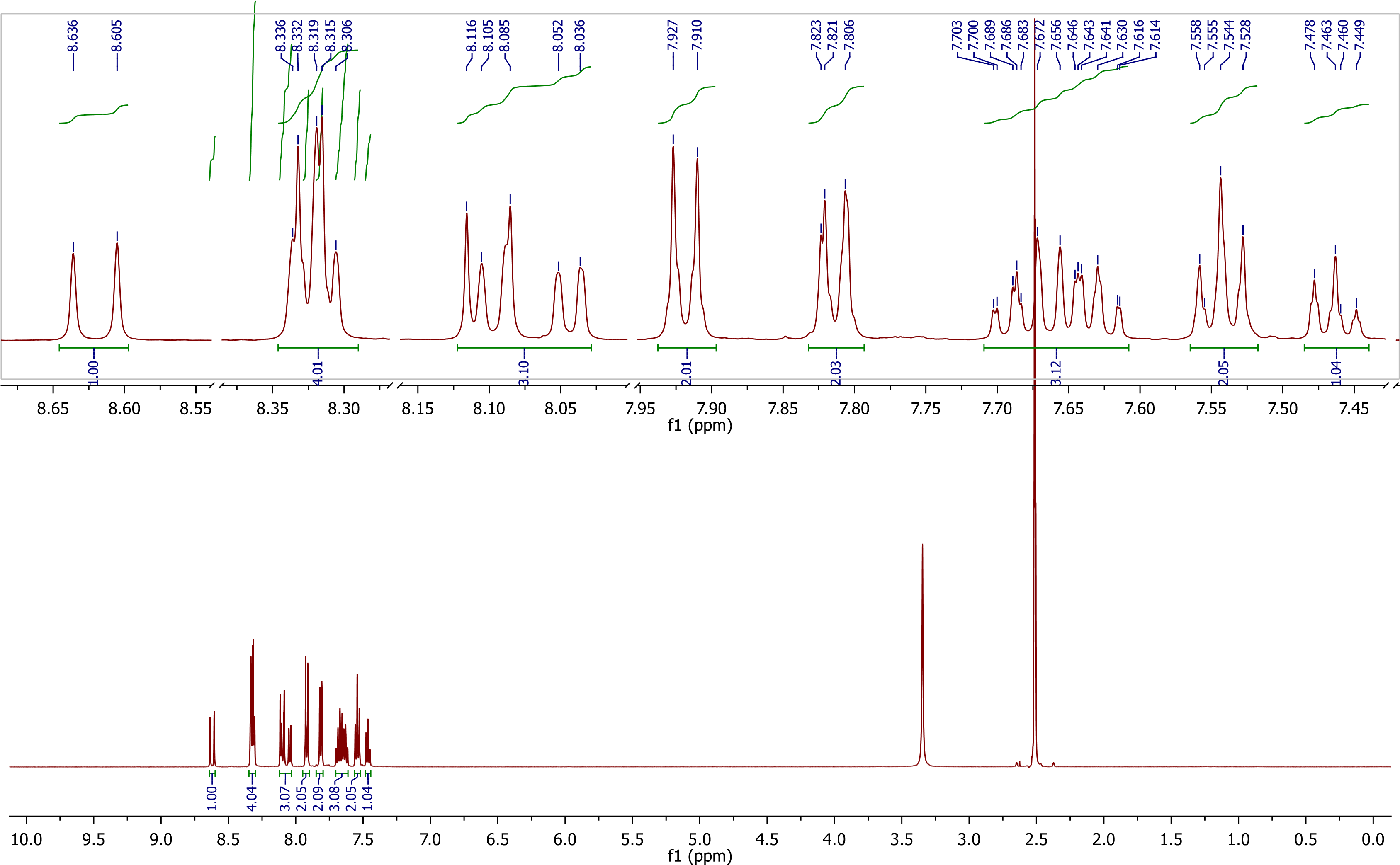


**Figure S22.** ^1^H NMR of 5-(1*H*-Imidazol-2-yl)-1-phenyl-3-(pyren-1-yl)-1*H*-pyrazole **(4h)**


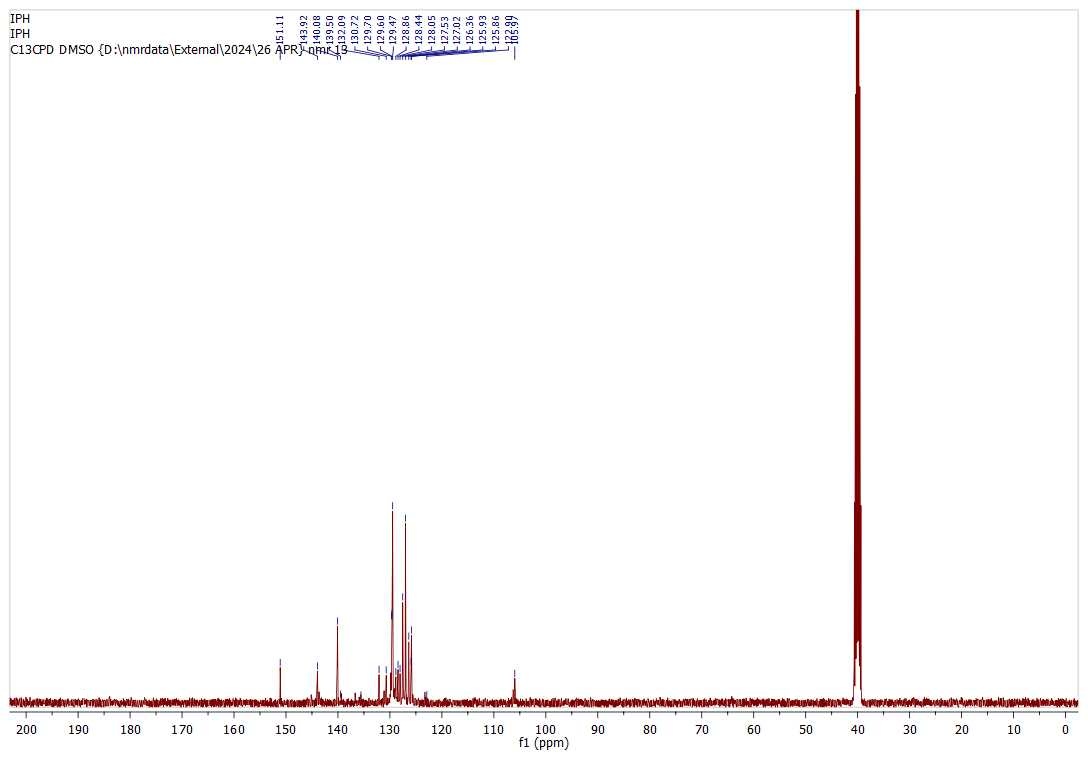


**Figure S23.** ^13^C NMR of 5-(1*H*-Imidazol-2-yl)-1-phenyl-3-(pyren-1-yl)-1*H*-pyrazole **(4h)**


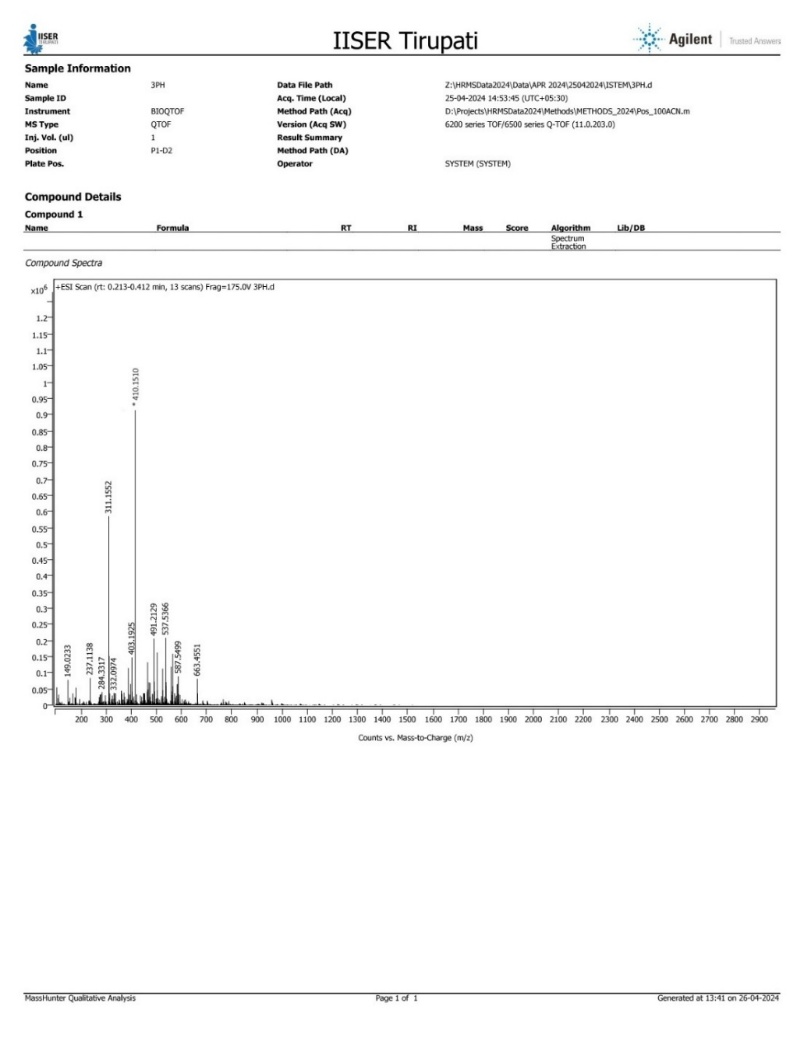


**Figure S24.** HRMS of 5-(1*H*-Imidazol-2-yl)-1-phenyl-3-(pyren-1-yl)-1*H*-pyrazole **(4h)**

**Pyrazole & protein Docked structure**

**
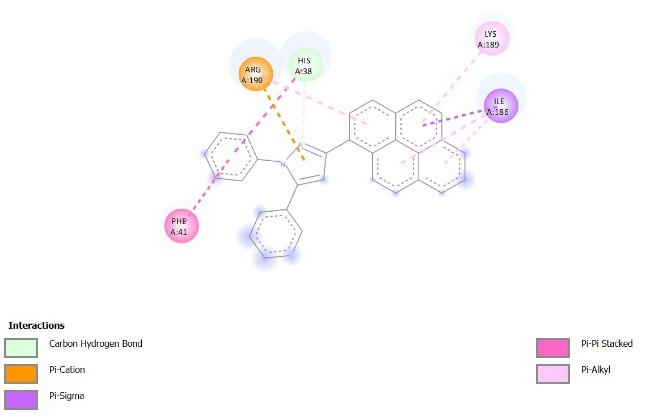
** **
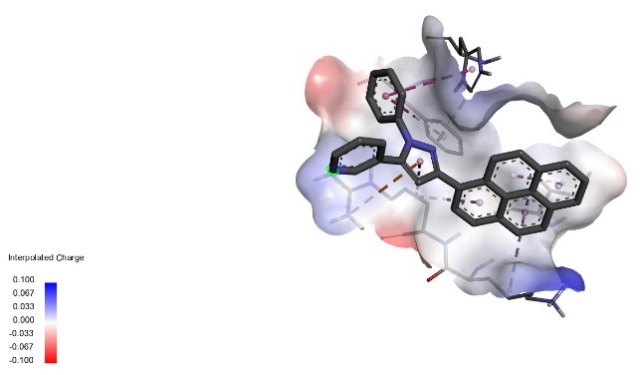
**

**Figure S25**. Two-dimensional (2D) and Three-dimensional (3D) orientations of docked Pyrazole **4a**

**
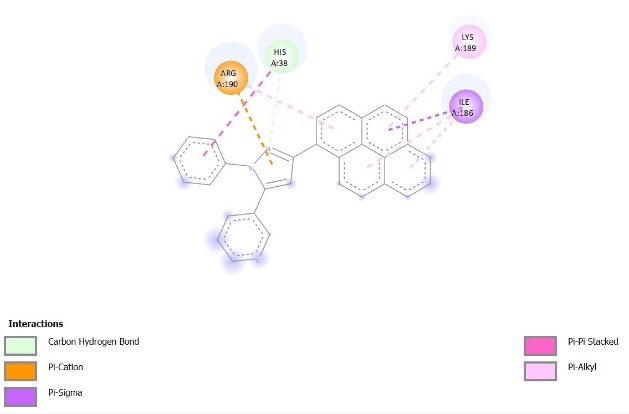
**  **
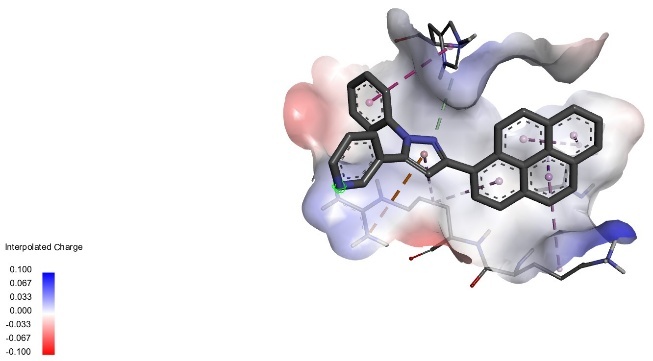
**

**Figure S26**. Two-dimensional (2D) and Three-dimensional (3D) orientations of docked Pyrazole **4b**

**
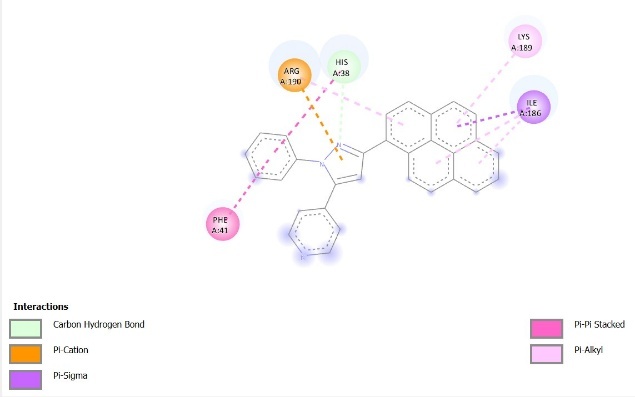
** **
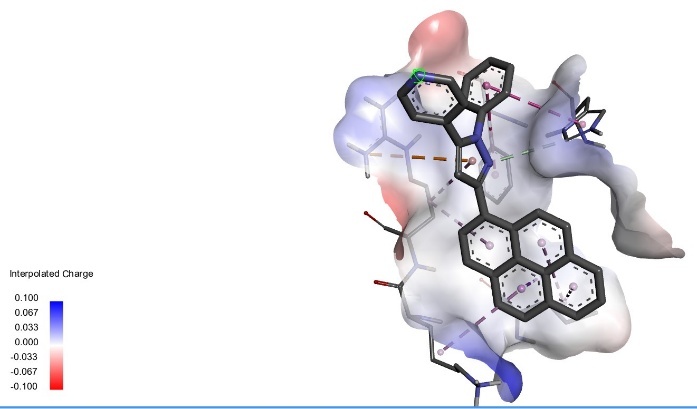
**

**Figure S27** Two-dimensional (2D) and Three-dimensional (3D) orientations of docked Pyrazole **4c**

**
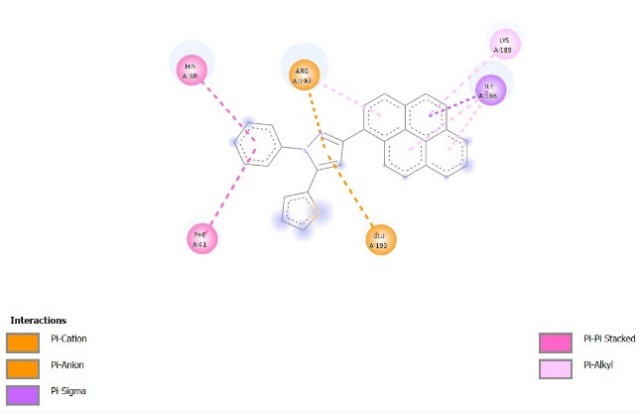
** **
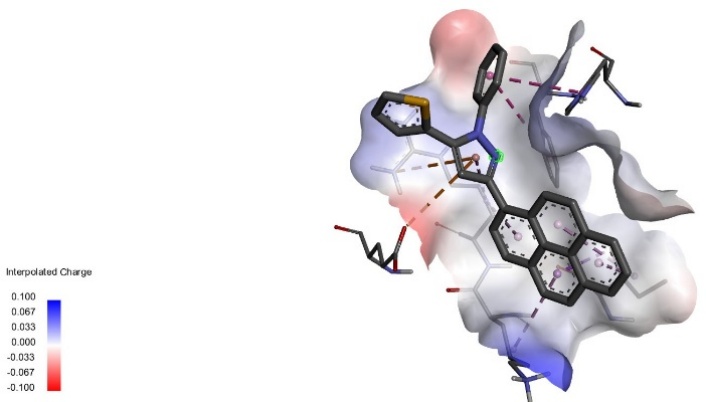
**

**Figure S28.** Two-dimensional (2D) and Three-dimensional (3D) orientations of docked Pyrazole **4d**

**
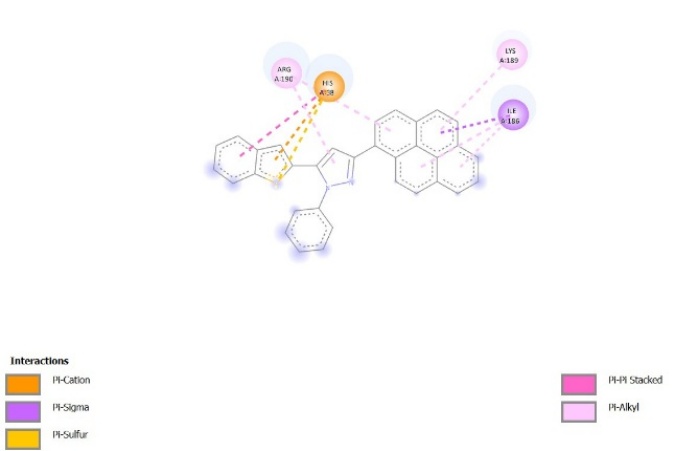

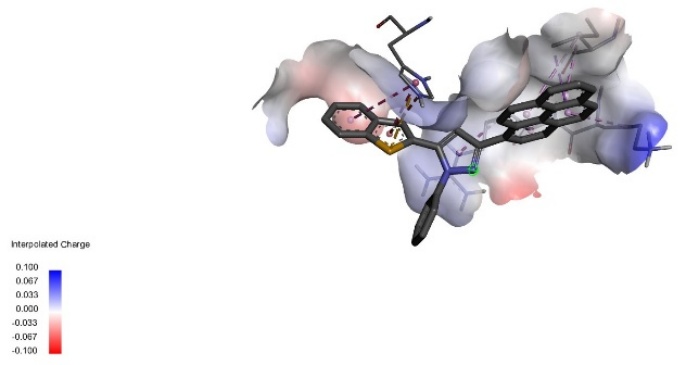
**

**Figure S29.** Two-dimensional (2D) and Three-dimensional (3D) orientations of docked Pyrazole **4e**

**
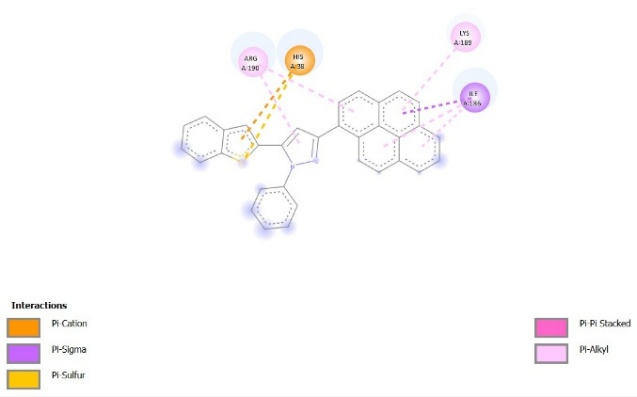

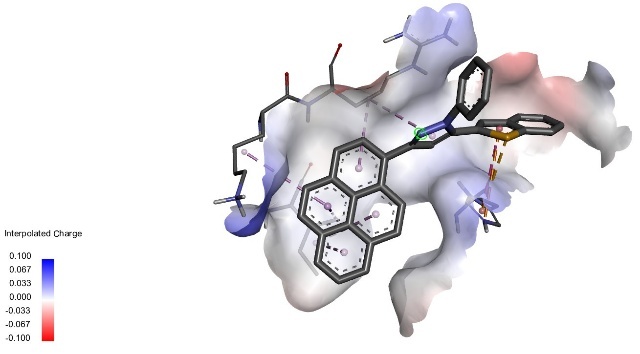
**

**Figure S30.** Two-dimensional (2D) and Three-dimensional (3D) orientations of docked Pyrazole **4f**

**
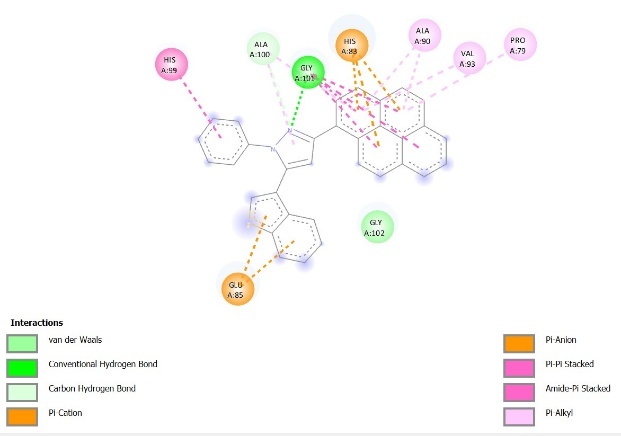

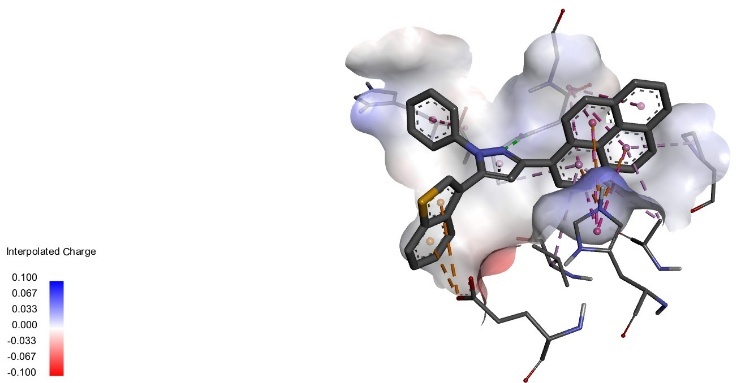
**

**Figure S31.** Two-dimensional (2D) and Three-dimensional (3D) orientations of docked Pyrazole **4g**

**Details of grid parameters**

**protein**

**spacing** 0.375

**npts**  40 40 40

**centre**  7.907 11.194 36.162

**Docking protocol parameters**

**Receptor**= protein. pdbqt

**Ligand**= ligand. pdbqt

**Centre_ X** = 40.043

**Centre_ Y** = -1.738

**Centre_ Z** = 45.797

**Size_ X**= 40

**Size_ Y** = 40

**Size_ Z** = 40

**Energy range** = 4

**Exhaustiveness** = 8

**Protein Information (PDBQT)**

REMARK 4 active torsions:

REMARK status: ('A' for Active; 'I' for Inactive)

REMARK 1 A between atoms: C_5 and C_7

REMARK 2 A between atoms: C_10 and C_13

REMARK 3 A between atoms: C_16 and C_24

REMARK 4 A between atoms: N_17 and C_18

**ROOT**

HETATM 1 C UNK 0 15.100 -9.552 0.156 0.00 0.00 0.001 A

HETATM 2 C UNK 0 14.670 -10.568 -0.695 0.00 0.00 0.000 A

HETATM 3 C UNK 0 15.584 -11.208 -1.527 0.00 0.00 0.001 A

HETATM 4 C UNK 0 16.931 -10.833 -1.507 0.00 0.00 0.012 A

HETATM 5 C UNK 0 17.386 -9.811 -0.657 0.00 0.00 -0.026 A

HETATM 6 C UNK 0 16.446 -9.177 0.173 0.00 0.00 0.012 A

**ENDROOT**

BRANCH 5 7

HETATM 7 C UNK 0 18.804 -9.416 -0.637 0.00 0.00 -0.026 A

HETATM 8 C UNK 0 19.818 -10.365 -0.436 0.00 0.00 0.013 A

HETATM 9 C UNK 0 21.169 -9.991 -0.414 0.00 0.00 0.014 A

HETATM 10 C UNK 0 21.548 -8.655 -0.594 0.00 0.00 -0.006 A

HETATM 11 C UNK 0 20.536 -7.705 -0.795 0.00 0.00 0.014 A

HETATM 12 C UNK 0 19.187 -8.079 -0.816 0.00 0.00 0.013 A

BRANCH 10 13

HETATM 13 C UNK 0 22.959 -8.248 -0.572 0.00 0.00 0.090 A

HETATM 14 N UNK 0 23.316 -6.963 -0.665 0.00 0.00 -0.166 NA

HETATM 15 N UNK 0 24.679 -6.929 -0.583 0.00 0.00 -0.231 N

HETATM 16 C UNK 0 25.201 -8.217 -0.527 0.00 0.00 0.090 A

HETATM 17 C UNK 0 24.107 -9.062 -0.476 0.00 0.00 0.068 A

BRANCH 15 18

HETATM 18 C UNK 0 25.314 -5.644 -0.503 0.00 0.00 0.059 A

HETATM 19 C UNK 0 26.647 -5.479 -0.108 0.00 0.00 0.031 A

HETATM 20 C UNK 0 27.212 -4.200 -0.030 0.00 0.00 0.002 A

HETATM 21 C UNK 0 26.452 -3.073 -0.336 0.00 0.00 0.000 A

HETATM 22 C UNK 0 25.122 -3.220 -0.715 0.00 0.00 0.002 A

HETATM 23 C UNK 0 24.555 -4.495 -0.793 0.00 0.00 0.031 A

ENDBRANCH 15 18

BRANCH 16 24

HETATM 24 C UNK 0 26.598 -8.603 -0.597 0.00 0.00 0.085 A

HETATM 25 C UNK 0 27.185 -9.404 0.377 0.00 0.00 0.034 A

HETATM 26 C UNK 0 28.519 -9.770 0.218 0.00 0.00 0.004 A

HETATM 27 C UNK 0 29.214 -9.339 -0.907 0.00 0.00 0.019 A

HETATM 28 C UNK 0 28.542 -8.556 -1.831 0.00 0.00 0.117 A

HETATM 29 N UNK 0 27.251 -8.182 -1.701 0.00 0.00 -0.259 NA

ENDBRANCH 16 24

ENDBRANCH 10 13

ENDBRANCH 5 7

TORSDOF 4

**Ligand Information files**

**MODEL 1**

REMARK VINA RESULT: -3.4 0.000 0.000

REMARK 4 active torsions:

REMARK status: ('A' for Active; 'I' for Inactive)

REMARK 1 A between atoms: C_5 and C_7

REMARK 2 A between atoms: C_10 and C_13

REMARK 3 A between atoms: C_15 and C_18

REMARK 4 A between atoms: N_16 and C_24

**ROOT**

HETATM 1 C UNK 0 44.861 -5.414 38.715 0.00 0.00 0.001 A

HETATM 2 C UNK 0 45.988 -5.943 39.342 0.00 0.00 0.000 A

HETATM 3 C UNK 0 46.439 -5.388 40.537 0.00 0.00 0.001 A

HETATM 4 C UNK 0 45.762 -4.305 41.105 0.00 0.00 0.012 A

HETATM 5 C UNK 0 44.625 -3.757 40.490 0.00 0.00 -0.026 A

HETATM 6 C UNK 0 44.187 -4.331 39.285 0.00 0.00 0.012 A

**ENDROOT**

BRANCH 5 7

HETATM 7 C UNK 0 43.913 -2.616 41.089 0.00 0.00 -0.026 A

HETATM 8 C UNK 0 43.174 -1.726 40.295 0.00 0.00 0.013 A

HETATM 9 C UNK 0 42.496 -0.637 40.861 0.00 0.00 0.014 A

HETATM 10 C UNK 0 42.537 -0.407 42.242 0.00 0.00 -0.006 A

HETATM 11 C UNK 0 43.273 -1.296 43.038 0.00 0.00 0.014 A

HETATM 12 C UNK 0 43.952 -2.382 42.470 0.00 0.00 0.013 A

BRANCH 10 13

HETATM 13 C UNK 0 41.832 0.729 42.850 0.00 0.00 0.090 A

HETATM 14 C UNK 0 41.288 0.832 44.146 0.00 0.00 0.066 A

HETATM 15 C UNK 0 40.724 2.092 44.244 0.00 0.00 0.072 A

HETATM 16 N UNK 0 40.968 2.712 43.019 0.00 0.00 -0.233 N

HETATM 17 N UNK 0 41.611 1.859 42.170 0.00 0.00 -0.166 NA

BRANCH 15 18

HETATM 18 C UNK 0 39.972 2.613 45.385 0.00 0.00 0.004 A

HETATM 19 C UNK 0 40.584 3.417 46.387 0.00 0.00 -0.013 A

HETATM 20 C UNK 0 39.792 3.857 47.478 0.00 0.00 -0.025 A

HETATM 21 C UNK 0 41.940 3.819 46.354 0.00 0.00 0.013 A

HETATM 22 C UNK 0 38.438 3.491 47.552 0.00 0.00 0.012 A

HETATM 23 C UNK 0 40.362 4.658 48.480 0.00 0.00 0.012 A

HETATM 24 C UNK 0 37.855 2.695 46.568 0.00 0.00 0.002 A

HETATM 25 C UNK 0 38.618 2.255 45.492 0.00 0.00 0.014 A

HETATM 26 C UNK 0 41.704 5.036 48.421 0.00 0.00 0.001 A

HETATM 27 C UNK 0 42.493 4.617 47.358 0.00 0.00 0.001 A

ENDBRANCH 15 18

BRANCH 16 28

HETATM 28 C UNK 0 40.663 4.033 42.549 0.00 0.00 0.059 A

HETATM 29 C UNK 0 41.607 5.065 42.501 0.00 0.00 0.031 A

HETATM 30 C UNK 0 41.250 6.336 42.034 0.00 0.00 0.002 A

HETATM 31 C UNK 0 39.950 6.591 41.604 0.00 0.00 0.000 A

HETATM 32 C UNK 0 39.003 5.574 41.636 0.00 0.00 0.002 A

HETATM 33 C UNK 0 39.356 4.305 42.101 0.00 0.00 0.031 A

ENDBRANCH 16 28

ENDBRANCH 10 13

ENDBRANCH 5 7

TORSDOF 4

ENDMDL

**MODEL 2**

REMARK VINA RESULT: -3.3 2.692 9.592

REMARK 4 active torsions:

REMARK status: ('A' for Active; 'I' for Inactive)

REMARK 1 A between atoms: C_5 and C_7

REMARK 2 A between atoms: C_10 and C_13

REMARK 3 A between atoms: C_15 and C_18

REMARK 4 A between atoms: N_16 and C_24

**ROOT**

HETATM 1 C UNK 0 36.452 5.416 50.803 0.00 0.00 0.001 A

HETATM 2 C UNK 0 35.213 5.786 50.284 0.00 0.00 0.000 A

HETATM 3 C UNK 0 34.946 5.596 48.930 0.00 0.00 0.001 A

HETATM 4 C UNK 0 35.918 5.035 48.098 0.00 0.00 0.012 A

HETATM 5 C UNK 0 37.173 4.655 48.601 0.00 0.00 -0.026 A

HETATM 6 C UNK 0 37.422 4.856 49.969 0.00 0.00 0.012 A

**ENDROOT**

BRANCH 5 7

HETATM 7 C UNK 0 38.197 4.065 47.723 0.00 0.00 -0.026 A

HETATM 8 C UNK 0 39.537 4.477 47.793 0.00 0.00 0.013 A

HETATM 9 C UNK 0 40.515 3.919 46.958 0.00 0.00 0.014 A

HETATM 10 C UNK 0 40.180 2.929 46.025 0.00 0.00 -0.006 A

HETATM 11 C UNK 0 38.843 2.513 45.954 0.00 0.00 0.014 A

HETATM 12 C UNK 0 37.867 3.074 46.788 0.00 0.00 0.013 A

BRANCH 10 13

HETATM 13 C UNK 0 41.193 2.335 45.143 0.00 0.00 0.090 A

HETATM 14 C UNK 0 41.142 1.109 44.449 0.00 0.00 0.066 A

HETATM 15 C UNK 0 42.334 0.991 43.758 0.00 0.00 0.072 A

HETATM 16 N UNK 0 43.051 2.154 44.038 0.00 0.00 -0.233 N

HETATM 17 N UNK 0 42.360 2.945 44.909 0.00 0.00 -0.166 NA

BRANCH 15 18

HETATM 18 C UNK 0 42.759 -0.161 42.963 0.00 0.00 0.004 A

HETATM 19 C UNK 0 43.554 -1.205 43.514 0.00 0.00 -0.013 A

HETATM 20 C UNK 0 43.949 -2.272 42.668 0.00 0.00 -0.025 A

HETATM 21 C UNK 0 43.965 -1.250 44.867 0.00 0.00 0.013 A

HETATM 22 C UNK 0 43.560 -2.276 41.319 0.00 0.00 0.012 A

HETATM 23 C UNK 0 44.728 -3.322 43.182 0.00 0.00 0.012 A

HETATM 24 C UNK 0 42.789 -1.242 40.791 0.00 0.00 0.002 A

HETATM 25 C UNK 0 42.393 -0.188 41.607 0.00 0.00 0.014 A

HETATM 26 C UNK 0 45.121 -3.337 44.520 0.00 0.00 0.001 A

HETATM 27 C UNK 0 44.739 -2.302 45.363 0.00 0.00 0.001 A

ENDBRANCH 15 18

BRANCH 16 28

HETATM 28 C UNK 0 44.328 2.614 43.572 0.00 0.00 0.059 A

HETATM 29 C UNK 0 45.477 2.620 44.370 0.00 0.00 0.031 A

HETATM 30 C UNK 0 46.697 3.082 43.860 0.00 0.00 0.002 A

HETATM 31 C UNK 0 46.785 3.540 42.548 0.00 0.00 0.000 A

HETATM 32 C UNK 0 45.655 3.532 41.738 0.00 0.00 0.002 A

HETATM 33 C UNK 0 44.437 3.070 42.244 0.00 0.00 0.031 A

ENDBRANCH 16 28

ENDBRANCH 10 13

ENDBRANCH 5 7

TORSDOF 4

ENDMDL

**MODEL 3**

REMARK VINA RESULT: -3.3 2.524 10.034

REMARK 4 active torsions:

REMARK status: ('A' for Active; 'I' for Inactive)

REMARK 1 A between atoms: C_ 5 and C_7

REMARK 2 A between atoms: C_10 and C_13

REMARK 3 A between atoms: C_15 and C_18

REMARK 4 A between atoms: N_16 and C_24

**ROOT**

HETATM 1 C UNK 0 39.534 4.431 47.215 0.00 0.00 0.001 A

HETATM 2 C UNK 0 40.429 5.391 47.681 0.00 0.00 0.000 A

HETATM 3 C UNK 0 41.777 5.303 47.341 0.00 0.00 0.001 A

HETATM 4 C UNK 0 42.227 4.254 46.534 0.00 0.00 0.012 A

HETATM 5 C UNK 0 41.341 3.276 46.054 0.00 0.00 -0.026 A

HETATM 6 C UNK 0 39.986 3.383 46.409 0.00 0.00 0.012 A

**ENDROOT**

BRANCH 5 7

HETATM 7 C UNK 0 41.817 2.172 45.203 0.00 0.00 -0.026 A

HETATM 8 C UNK 0 43.161 1.771 45.222 0.00 0.00 0.013 A

HETATM 9 C UNK 0 43.618 0.721 44.413 0.00 0.00 0.014 A

HETATM 10 C UNK 0 42.741 0.039 43.560 0.00 0.00 -0.006 A

HETATM 11 C UNK 0 41.397 0.436 43.541 0.00 0.00 0.014 A

HETATM 12 C UNK 0 40.943 1.487 44.349 0.00 0.00 0.013 A

BRANCH 10 13

HETATM 13 C UNK 0 43.205 -1.062 42.706 0.00 0.00 0.090 A

HETATM 14 C UNK 0 44.006 -2.167 43.058 0.00 0.00 0.066 A

HETATM 15 C UNK 0 44.173 -2.921 41.911 0.00 0.00 0.072 A

HETATM 16 N UNK 0 43.452 -2.262 40.916 0.00 0.00 -0.233 N

HETATM 17 N UNK 0 42.898 -1.115 41.406 0.00 0.00 -0.166 NA

BRANCH 15 18

HETATM 18 C UNK 0 45.001 -4.120 41.780 0.00 0.00 0.004 A

HETATM 19 C UNK 0 44.451 -5.429 41.875 0.00 0.00 -0.013 A

HETATM 20 C UNK 0 45.326 -6.540 41.769 0.00 0.00 -0.025 A

HETATM 21 C UNK 0 43.073 -5.693 42.056 0.00 0.00 0.013 A

HETATM 22 C UNK 0 46.702 -6.330 41.580 0.00 0.00 0.012 A

HETATM 23 C UNK 0 44.815 -7.845 41.850 0.00 0.00 0.012 A

HETATM 24 C UNK 0 47.228 -5.043 41.499 0.00 0.00 0.002 A

HETATM 25 C UNK 0 46.383 -3.943 41.602 0.00 0.00 0.014 A

HETATM 26 C UNK 0 43.451 -8.073 42.032 0.00 0.00 0.001 A

HETATM 27 C UNK 0 42.580 -6.997 42.135 0.00 0.00 0.001 A

ENDBRANCH 15 18

BRANCH 16 28

HETATM 28 C UNK 0 43.218 -2.593 39.539 0.00 0.00 0.059 A

HETATM 29 C UNK 0 42.183 -3.435 39.120 0.00 0.00 0.031 A

HETATM 30 C UNK 0 42.004 -3.722 37.760 0.00 0.00 0.002 A

HETATM 31 C UNK 0 42.852 -3.169 36.805 0.00 0.00 0.000 A

HETATM 32 C UNK 0 43.879 -2.321 37.204 0.00 0.00 0.002 A

HETATM 33 C UNK 0 44.060 -2.033 38.559 0.00 0.00 0.031 A

ENDBRANCH 16 28

ENDBRANCH 10 13

ENDBRANCH 5 7

TORSDOF 4

ENDMDL

**MODEL 4**

REMARK VINA RESULT: -3.3 2.433 9.615

REMARK 4 active torsions:

REMARK status: ('A' for Active; 'I' for Inactive)

REMARK 1 A between atoms: C_5 and C_7

REMARK 2 A between atoms: C_10 and C_13

REMARK 3 A between atoms: C_15 and C_18

REMARK 4 A between atoms: N_16 and C_24

**ROOT**

HETATM 1 C UNK 0 37.837 6.328 48.080 0.00 0.00 0.001 A

HETATM 2 C UNK 0 37.674 5.910 49.399 0.00 0.00 0.000 A

HETATM 3 C UNK 0 38.289 4.738 49.834 0.00 0.00 0.001 A

HETATM 4 C UNK 0 39.067 3.986 48.950 0.00 0.00 0.012 A

HETATM 5 C UNK 0 39.244 4.390 47.616 0.00 0.00 -0.026 A

HETATM 6 C UNK 0 38.615 5.574 47.198 0.00 0.00 0.012 A

**ENDROOT**

BRANCH 5 7

HETATM 7 C UNK 0 40.064 3.597 46.685 0.00 0.00 -0.026 A

HETATM 8 C UNK 0 41.443 3.822 46.561 0.00 0.00 0.013 A

HETATM 9 C UNK 0 42.227 3.071 45.675 0.00 0.00 0.014 A

HETATM 10 C UNK 0 41.652 2.068 44.884 0.00 0.00 -0.006 A

HETATM 11 C UNK 0 40.274 1.839 45.007 0.00 0.00 0.014 A

HETATM 12 C UNK 0 39.492 2.593 45.892 0.00 0.00 0.013 A

BRANCH 10 13

HETATM 13 C UNK 0 42.460 1.272 43.950 0.00 0.00 0.090 A

HETATM 14 C UNK 0 42.245 -0.050 43.512 0.00 0.00 0.066 A

HETATM 15 C UNK 0 43.277 -0.355 42.643 0.00 0.00 0.072 A

HETATM 16 N UNK 0 44.062 0.795 42.568 0.00 0.00 -0.233 N

HETATM 17 N UNK 0 43.572 1.764 43.395 0.00 0.00 -0.166 NA

BRANCH 15 18

HETATM 18 C UNK 0 43.512 -1.650 42.004 0.00 0.00 0.004 A

HETATM 19 C UNK 0 43.146 -1.899 40.651 0.00 0.00 -0.013 A

HETATM 20 C UNK 0 43.379 -3.192 40.116 0.00 0.00 -0.025 A

HETATM 21 C UNK 0 42.573 -0.927 39.798 0.00 0.00 0.013 A

HETATM 22 C UNK 0 43.953 -4.189 40.921 0.00 0.00 0.012 A

HETATM 23 C UNK 0 43.037 -3.470 38.783 0.00 0.00 0.012 A

HETATM 24 C UNK 0 44.297 -3.933 42.246 0.00 0.00 0.002 A

HETATM 25 C UNK 0 44.074 -2.671 42.787 0.00 0.00 0.014 A

HETATM 26 C UNK 0 42.470 -2.490 37.967 0.00 0 .00 0.001 A

HETATM 27 C UNK 0 42.239 -1.219 38.474 0.00 0.00 0.001 A

ENDBRANCH 15 18

BRANCH 16 28

HETATM 28 C UNK 0 45.234 1.093 41.796 0.00 0.00 0.059 A

HETATM 29 C UNK 0 46.530 0.773 42.216 0.00 0.00 0.031 A

HETATM 30 C UNK 0 47.637 1.090 41.419 0.00 0.00 0.002 A

HETATM 31 C UNK 0 47.464 1.725 40.192 0.00 0.00 0.000 A

HETATM 32 C UNK 0 46.183 2.041 39.755 0.00 0.00 0.002 A

HETATM 33 C UNK 0 45.077 1.726 40.547 0.00 0.00 0.031 A

ENDBRANCH 16 28

ENDBRANCH 10 13

ENDBRANCH 5 7

TORSDOF 4

ENDMDL

**MODEL 5**

REMARK VINA RESULT: -3.2 3.043 9.987

REMARK 4 active torsions:

REMARK status: ('A' for Active; 'I' for Inactive)

REMARK 1 A between atoms: C_5 and C_7

REMARK 2 A between atoms: C_10 and C_13

REMARK 3 A between atoms: C_15 and C_18

REMARK 4 A between atoms: N_16 and C_24

**ROOT**

HETATM 1 C UNK 0 41.148 4.519 47.612 0.00 0.00 0.001 A

HETATM 2 C UNK 0 39.964 4.444 48.343 0.00 0.00 0.000 A

HETATM 3 C UNK 0 38.927 3.628 47.899 0.00 0.00 0.001 A

HETATM 4 C UNK 0 39.076 2.886 46.724 0.00 0.00 0.012 A

HETATM 5 C UNK 0 40.261 2.946 45.972 0.00 0.00 -0.026 A

HETATM 6 C UNK 0 41.294 3.777 46.438 0.00 0.00 0.012 A

**ENDROOT**

BRANCH 5 7

HETATM 7 C UNK 0 40.417 2.165 44.734 0.00 0.00 -0.026 A

HETATM 8 C UNK 0 41.523 1.323 44.543 0.00 0.00 0.013 A

HETATM 9 C UNK 0 41.676 0.578 43.366 0.00 0.00 0.014 A

HETATM 10 C UNK 0 40.723 0.653 42.342 0.00 0.00 -0.006 A

HETATM 11 C UNK 0 39.616 1.492 42.530 0.00 0.00 0.014 A

HETATM 12 C UNK 0 39.466 2.237 43.707 0.00 0.00 0.013 A

BRANCH 10 13

HETATM 13 C UNK 0 40.870 -0.121 41.102 0.00 0.00 0.090 A

HETATM 14 C UNK 0 42.048 -0.630 40.520 0.00 0.00 0.066 A

HETATM 15 C UNK 0 41.671 -1.281 39.359 0.00 0.00 0.072 A

HETATM 16 N UNK 0 40.283 -1.164 39.292 0.00 0.00 -0.233 N

HETATM 17 N UNK 0 39.814 -0.426 40.340 0.00 0.00 -0.166 NA

BRANCH 15 18

HETATM 18 C UNK 0 42.573 -1.895 38.384 0.00 0.00 0.004 A

HETATM 19 C UNK 0 42.815 -3.297 38.362 0.00 0.00 -0.013 A

HETATM 20 C UNK 0 43.727 -3.810 37.404 0.00 0.00 -0.025 A

HETATM 21 C UNK 0 42.188 -4.218 39.233 0.00 0.00 0.013 A

HETATM 22 C UNK 0 44.370 -2.936 36.512 0.00 0.00 0.012 A

HETATM 23 C UNK 0 43.984 -5.189 37.350 0.00 0.00 0.012 A

HETATM 24 C UNK 0 44.130 -1.565 36.552 0.00 0.00 0.002 A

HETATM 25 C UNK 0 43.239 -1.045 37.485 0.00 0.00 0.014 A

HETATM 26 C UNK 0 43.352 -6.073 38.225 0.00 0.00 0.001 A

HETATM 27 C UNK 0 42.454 -5.588 39.166 0.00 0.00 0.001 A

ENDBRANCH 15 18

BRANCH 16 28

HETATM 28 C UNK 0 39.334 -1.675 38.345 0.00 0.00 0.059 A

HETATM 29 C UNK 0 39.011 -1.015 37.154 0.00 0.00 0.031 A

HETATM 30 C UNK 0 38.075 -1.564 36.268 0.00 0.00 0.002 A

HETATM 31 C UNK 0 37.456 -2.777 36.556 0.00 0.00 0.000 A

HETATM 32 C UNK 0 37.774 -3.450 37.730 0.00 0.00 0.002 A

HETATM 33 C UNK 0 38.707 -2.906 38.616 0.00 0.00 0.031 A

ENDBRANCH 16 28

ENDBRANCH 10 13

ENDBRANCH 5 7

TORSDOF 4

ENDMDL

**MODEL 6**

REMARK VINA RESULT: -3.2 3.506 10.061

REMARK 4 active torsions:

REMARK status: ('A' for Active; 'I' for Inactive)

REMARK 1 A between atoms: C_5 and C_7

REMARK 2 A between atoms: C_10 and C_13

REMARK 3 A between atoms: C_15 and C_18

REMARK 4 A between atoms: N_16 and C_24

**ROOT**

HETATM 1 C UNK 0 40.946 4.455 47.667 0.00 0.00 0.001 A

HETATM 2 C UNK 0 39.758 4.353 48.388 0.00 0.00 0.000 A

HETATM 3 C UNK 0 38.732 3.536 47.920 0.00 0.00 0.001 A

HETATM 4 C UNK 0 38.896 2.820 46.731 0.00 0.00 0.012 A

HETATM 5 C UNK 0 40.085 2.908 45.988 0.00 0.00 -0.026 A

HETATM 6 C UNK 0 41.107 3.738 46.478 0.00 0.00 0.012 A

**ENDROOT**

BRANCH 5 7

HETATM 7 C UNK 0 40.256 2.153 44.735 0.00 0.00 -0.026 A

HETATM 8 C UNK 0 41.366 1.318 44.539 0.00 0.00 0.013 A

HETATM 9 C UNK 0 41.534 0.599 43.347 0.00 0.00 0.014 A

HETATM 10 C UNK 0 40.593 0.695 42.314 0.00 0.00 -0.006 A

HETATM 11 C UNK 0 39.482 1.528 42.508 0.00 0.00 0.014 A

HETATM 12 C UNK 0 39.318 2.247 43.699 0.00 0.00 0.013 A

BRANCH 10 13

HETATM 13 C UNK 0 40.755 -0.051 41.059 0.00 0.00 0.090 A

HETATM 14 C UNK 0 39.828 -0.215 40.011 0.00 0.00 0.066 A

HETATM 15 C UNK 0 40.443 -1.002 39.054 0.00 0.00 0.072 A

HETATM 16 N UNK 0 41.721 -1.265 39.543 0.00 0.00 -0.233 N

HETATM 17 N UNK 0 41.884 -0.710 40.779 0.00 0.00 -0.166 NA

BRANCH 15 18

HETATM 18 C UNK 0 39.828 -1.500 37.823 0.00 0.00 0.004 A

HETATM 19 C UNK 0 39.230 -2.789 37.748 0.00 0.00 -0.013 A

HETATM 20 C UNK 0 38.683 -3.209 36.509 0.00 0.00 -0.025 A

HETATM 21 C UNK 0 39.135 -3.678 38.845 0.00 0.00 0.013 A

HETATM 22 C UNK 0 38.745 -2.360 35.393 0.00 0.00 0.012 A

HETATM 23 C UNK 0 38.078 -4.473 36.403 0.00 0.00 0.012 A

HETATM 24 C UNK 0 39.343 -1.104 35.479 0.00 0.00 0.002 A

HETATM 25 C UNK 0 39.884 -0.677 36.686 0.00 0.00 0.014 A

HETATM 26 C UNK 0 38.003 -5.328 37.501 0.00 0.00 0.001 A

HETATM 27 C UNK 0 38.530 -4.931 38.723 0.00 0.00 0.001 A

ENDBRANCH 15 18

BRANCH 16 28

HETATM 28 C UNK 0 42.823 -1.987 38.974 0.00 0.00 0.059 A

HETATM 29 C UNK 0 43.868 -1.366 38.281 0.00 0.00 0.031 A

HETATM 30 C UNK 0 44.918 -2.123 37.746 0.00 0.00 0.002 A

HETATM 31 C UNK 0 44.940 -3.507 37.901 0.00 0.00 0.000 A

HETATM 32 C UNK 0 43.915 -4.138 38.597 0.00 0.00 0.002 A

HETATM 33 C UNK 0 42.867 -3.386 39.132 0.00 0.00 0.031 A

ENDBRANCH 16 28

ENDBRANCH 10 13

ENDBRANCH 5 7

TORSDOF 4

ENDMDL

**MODEL 7**

REMARK VINA RESULT: -3.2 6.393 8.034

REMARK 4 active torsions:

REMARK status: ('A' for Active; 'I' for Inactive)

REMARK 1 A between atoms: C_5 and C_7

REMARK 2 A between atoms: C_10 and C_13

REMARK 3 A between atoms: C_15 and C_18

REMARK 4 A between atoms: N_16 and C_24

**ROOT**

HETATM 1 C UNK 0 54.491 2.148 41.185 0.00 0.00 0.001 A

HETATM 2 C UNK 0 55.499 2.304 42.134 0.00 0.00 0.000 A

HETATM 3 C UNK 0 55.193 2.207 43.489 0.00 0.00 0.001 A

HETATM 4 C UNK 0 53.880 1.953 43.894 0.00 0.00 0.012 A

HETATM 5 C UNK 0 52.849 1.793 42.953 0.00 0.00 -0.026 A

HETATM 6 C UNK 0 53.178 1.895 41.592 0.00 0.00 0.012 A

**ENDROOT**

BRANCH 5 7

HETATM 7 C UNK 0 51.465 1.526 43.381 0.00 0.00 -0.026 A

HETATM 8 C UNK 0 50.392 1.665 42.488 0.00 0.00 0.013 A

HETATM 9 C UNK 0 49.073 1.414 42.891 0.00 0.00 0.014 A

HETATM 10 C UNK 0 48.788 1.014 44.203 0.00 0.00 -0.006 A

HETATM 11 C UNK 0 49.859 0.872 45.097 0.00 0.00 0.014 A

HETATM 12 C UNK 0 51.176 1.126 44.692 0.00 0.00 0.013 A

BRANCH 10 13

HETATM 13 C UNK 0 47.412 0.748 44.640 0.00 0.00 0.090 A

HETATM 14 C UNK 0 46.206 1.187 44.058 0.00 0.00 0.066 A

HETATM 15 C UNK 0 45.180 0.681 44.835 0.00 0.00 0.072 A

HETATM 16 N UNK 0 45.797 -0.063 45.840 0.00 0.00 -0.233 N

HETATM 17 N UNK 0 47.154 0.016 45.729 0.00 0.00 -0.166 NA

BRANCH 15 18

HETATM 18 C UNK 0 43.752 0.947 44.656 0.00 0.00 0.004 A

HETATM 19 C UNK 0 43.093 2.007 45.338 0.00 0.00 -0.013 A

HETATM 20 C UNK 0 41.700 2.179 45.134 0.00 0.00 -0.025 A

HETATM 21 C UNK 0 43.748 2.915 46.203 0.00 0.00 0.013 A

HETATM 22 C UNK 0 41.006 1.308 44.279 0.00 0.00 0.012 A

HETATM 23 C UNK 0 41.020 3.221 45.785 0.00 0.00 0.012 A

HETATM 24 C UNK 0 41.663 0.268 43.625 0.00 0.00 0.002 A

HETATM 25 C UNK 0 43.028 0.086 43.815 0.00 0.00 0.014 A

HETATM 26 C UNK 0 41.693 4.098 46.635 0.00 0.00 0.001 A

HETATM 27 C UNK 0 43.057 3.946 46.843 0.00 0.00 0.001 A

ENDBRANCH 15 18

BRANCH 16 28

HETATM 28 C UNK 0 45.248 -0.853 46.905 0.00 0.00 0.059 A

HETATM 29 C UNK 0 44.556 -2.050 46.693 0.00 0.00 0.031 A

HETATM 30 C UNK 0 44.042 -2.777 47.774 0.00 0.00 0.002 A

HETATM 31 C UNK 0 44.218 -2.322 49.078 0.00 0.00 0.000 A

HETATM 32 C UNK 0 44.912 -1.140 49.307 0.00 0.00 0.002 A

HETATM 33 C UNK 0 45.426 -0.412 48.231 0.00 0.00 0.031 A

ENDBRANCH 16 28

ENDBRANCH 10 13

ENDBRANCH 5 7

TORSDOF 4

ENDMDL

**MODEL 8**

REMARK VINA RESULT: -3.2 2.420 4.301

REMARK 4 active torsions:

REMARK status: ('A' for Active; 'I' for Inactive)

REMARK 1 A between atoms: C_5 and C_7

REMARK 2 A between atoms: C_10 and C_13

REMARK 3 A between atoms: C_15 and C_18

REMARK 4 A between atoms: N_16 and C_24

**ROOT**

HETATM 1 C UNK 0 44.747 -3.700 41.780 0.00 0.00 0.001 A

HETATM 2 C UNK 0 44.628 -3.620 40.395 0.00 0.00 0.000 A

HETATM 3 C UNK 0 43.930 -2.560 39.820 0.00 0.00 0.001 A

HETATM 4 C UNK 0 43.353 -1.580 40.633 0.00 0.00 0.012 A

HETATM 5 C UNK 0 43.461 -1.642 42.031 0.00 0.00 -0.026 A

HETATM 6 C UNK 0 44.169 -2.719 42.591 0.00 0.00 0.012 A

**ENDROOT**

BRANCH 5 7

HETATM 7 C UNK 0 42.853 -0.609 42.887 0.00 0.00 -0.026 A

HETATM 8 C UNK 0 41.639 0.001 42.534 0.00 0.00 0.013 A

HETATM 9 C UNK 0 41.057 0.985 43.344 0.00 0.00 0.014 A

HETATM 10 C UNK 0 41.674 1.390 44.536 0.00 0.00 -0.006 A

HETATM 11 C UNK 0 42.886 0.781 44.891 0.00 0.00 0.014 A

HETATM 12 C UNK 0 43.467 -0.201 44.078 0.00 0.00 0.013 A

BRANCH 10 13

HETATM 13 C UNK 0 41.077 2.421 45.395 0.00 0.00 0.090 A

HETATM 14 C UNK 0 39.729 2.571 45.778 0.00 0.00 0.066 A

HETATM 15 C UNK 0 39.666 3.691 46.586 0.00 0.00 0.072 A

HETATM 16 N UNK 0 40.974 4.163 46.683 0.00 0.00 -0.233 N

HETATM 17 N UNK 0 41.815 3.402 45.925 0.00 0.00 -0.166 NA

BRANCH 15 18

HETATM 18 C UNK 0 38.450 4.278 47.151 0.00 0.00 0.004 A

HETATM 19 C UNK 0 38.091 4.096 48.516 0.00 0.00 -0.013 A

HETATM 20 C UNK 0 36.880 4.672 48.978 0.00 0.00 -0.025 A

HETATM 21 C UNK 0 38.878 3.382 49.449 0.00 0.00 0.013 A

HETATM 22 C UNK 0 36.067 5.397 48.092 0.00 0.00 0.012 A

HETATM 23 C UNK 0 36.499 4.518 50.321 0.00 0.00 0.012 A

HETATM 24 C UNK 0 36.428 5.559 46.756 0.00 0.00 0.002 A

HETATM 25 C UNK 0 37.611 5.000 46.287 0.00 0.00 0.014 A

HETATM 26 C UNK 0 37.295 3.805 51.217 0.00 0.00 0.001 A

HETATM 27 C UNK 0 38.485 3.237 50.782 0.00 0.00 0.001 A

ENDBRANCH 15 18

BRANCH 16 28

HETATM 28 C UNK 0 41.532 5.262 47.418 0.00 0.00 0.059 A

HETATM 29 C UNK 0 41.481 6.587 46.970 0.00 0.00 0.031 A

HETATM 30 C UNK 0 42.045 7.618 47.731 0.00 0.00 0.002 A

HETATM 31 C UNK 0 42.661 7.342 48.949 0.00 0.00 0.000 A

HETATM 32 C UNK 0 42.711 6.032 49.413 0.00 0.00 0.002 A

HETATM 33 C UNK 0 42.149 5.001 48.657 0.00 0.00 0.031 A

ENDBRANCH 16 28

ENDBRANCH 10 13

ENDBRANCH 5 7

TORSDOF 4

ENDMDL

**MODEL 9**

REMARK VINA RESULT: -3.2 3.088 5.752

REMARK 4 active torsions:

REMARK status: ('A' for Active; 'I' for Inactive)

REMARK 1 A between atoms: C_5 and C_7

REMARK 2 A between atoms: C_10 and C_13

REMARK 3 A between atoms: C_15 and C_18

REMARK 4 A between atoms: N_16 and C_24

**ROOT**

HETATM 1 C UNK 0 40.239 -0.962 39.236 0.00 0.00 0.001 A

HETATM 2 C UNK 0 41.523 -1.441 38.988 0.00 0.00 0.000 A

HETATM 3 C UNK 0 42.543 -1.194 39.904 0.00 0.00 0.001 A

HETATM 4 C UNK 0 42.276 -0.470 41.068 0.00 0.00 0.012 A

HETATM 5 C UNK 0 40.988 0.022 41.338 0.00 0.00 -0.026 A

HETATM 6 C UNK 0 39.974 -0.238 40.401 0.00 0.00 0.012 A

**ENDROOT**

BRANCH 5 7

HETATM 7 C UNK 0 40.708 0.785 42.566 0.00 0.00 -0.026 A

HETATM 8 C UNK 0 41.634 0.831 43.618 0.00 0.00 0.013 A

HETATM 9 C UNK 0 41.371 1.555 44.789 0.00 0.00 0.014 A

HETATM 10 C UNK 0 40.169 2.259 44.941 0.00 0.00 -0.006 A

HETATM 11 C UNK 0 39.242 2.217 43.891 0.00 0.00 0.014 A

HETATM 12 C UNK 0 39.507 1.490 42.722 0.00 0.00 0.013 A

BRANCH 10 13

HETATM 13 C UNK 0 39.879 3.022 46.162 0.00 0.00 0.090 A

HETATM 14 C UNK 0 38.721 2.985 46.964 0.00 0.00 0.066 A

HETATM 15 C UNK 0 38.920 3.884 47.996 0.00 0.00 0.072 A

HETATM 16 N UNK 0 40.195 4.413 47.796 0.00 0.00 -0.233 N

HETATM 17 N UNK 0 40.751 3.905 46.658 0.00 0.00 -0.166 NA

BRANCH 15 18

HETATM 18 C UNK 0 37.948 4.240 49.030 0.00 0.00 0.004 A

HETATM 19 C UNK 0 37.972 3.645 50.322 0.00 0.00 -0.013 A

HETATM 20 C UNK 0 36.974 4.022 51.257 0.00 0.00 -0.025 A

HETATM 21 C UNK 0 38.943 2.706 50.739 0.00 0.00 0.013 A

HETATM 22 C UNK 0 35.992 4.957 50.894 0.00 0.00 0.012 A

HETATM 23 C UNK 0 36.973 3.460 52.545 0.00 0.00 0.012 A

HETATM 24 C UNK 0 35.976 5.524 49.622 0.00 0.00 0.002 A

HETATM 25 C UNK 0 36.946 5.165 48.692 0.00 0.00 0.014 A

HETATM 26 C UNK 0 37.943 2.533 52.925 0.00 0.00 0.001 A

HETATM 27 C UNK 0 38.929 2.156 52.023 0.00 0.00 0.001 A

ENDBRANCH 15 18

BRANCH 16 28

HETATM 28 C UNK 0 40.962 5.352 48.564 0.00 0.00 0.059 A

HETATM 29 C UNK 0 40.865 6.737 48.396 0.00 0.00 0.031 A

HETATM 30 C UNK 0 41.641 7.605 49.176 0.00 0.00 0.002 A

HETATM 31 C UNK 0 42.516 7.102 50.134 0.00 0.00 0.000 A

HETATM 32 C UNK 0 42.616 5.728 50.321 0.00 0.00 0.002 A

HETATM 33 C UNK 0 41.844 4.860 49.546 0.00 0.00 0.031 A

ENDBRANCH 16 28

ENDBRANCH 10 13

ENDBRANCH 5 7

TORSDOF 4

ENDMDL
